# Metabolite changes in conifer buds and needles during forced bud break in Norway spruce (*Picea abies*) and European silver fir (*Abies alba*)

**DOI:** 10.3389/fpls.2014.00706

**Published:** 2014-12-11

**Authors:** Priyanka Dhuli, Jens Rohloff, G. Richard Strimbeck

**Affiliations:** Department of Biology, Norwegian University of Science and Technology (NTNU)Trondheim, Norway

**Keywords:** climate change, cold acclimation, dormancy, dehardening, bud break, GC/MS, metabolite profiling, Pinaceae

## Abstract

Environmental changes such as early spring and warm spells induce bud burst and photosynthetic processes in cold-acclimated coniferous trees and consequently, cellular metabolism in overwintering needles and buds. The purpose of the study was to examine metabolism in conifers under forced deacclimation (artificially induced spring) by exposing shoots of *Picea abies* (boreal species) and *Abies alba* (temperate species) to a greenhouse environment (22°C, 16/8 h D/N cycle) over a 9 weeks period. Each week, we scored bud opening and collected samples for GC/MS–based metabolite profiling. We detected a total of 169 assigned metabolites and 80 identified metabolites, comprising compounds such as mono- and disaccharides, Krebs cycle acids, amino acids, polyols, phenolics, and phosphorylated structures. Untargeted multivariate statistical analysis based on PCA and cluster analysis segregated samples by species, tissue type, and stage of tissue deacclimations. Similar patterns of metabolic regulation in both species were observed in buds (amino acids, Krebs cycle acids) and needles (hexoses, pentoses, and Krebs cycle acids). Based on correlation of bud opening score with compound levels, distinct metabolites could be associated with bud and shoot development, including amino acids, sugars, and acids with known osmolyte function, and secondary metabolites. This study has shed light on how elevated temperature affects metabolism in buds and needles of conifer species during the deacclimation phase, and contributes to the discussion about how phenological characters in conifers may respond to future global warming.

## Introduction

Over the past 150 years, burning of carbon-rich petroleum fuel and industrialization have resulted in an approximately 43% increase in CO_2_ concentration, from a preindustrial concentration of about 280 ppm to nearly 400 ppm today (IPCC, 2007). This has resulted in well-documented increases in global temperature and associated climate patterns, with warming generally greater at higher latitudes. These trends are expected to continue. One possible consequence of global warming is disruption of the phenology of bud set, dormancy, and bud break in trees and other plants in cold-temperate and boreal forest ecosystems.

Globally, forests represent the largest terrestrial carbon pool on earth, most of which is stored in northern boreal woody forests (Dixon et al., 1994). Coniferous and mixed forests cover an area of about 2.5 × 10^6^ km^2^ and act as a carbon sinks (Breymeyer et al., 1996). Investigation of the effects of climate warming on some boreal conifers has revealed alterations in carbon balance between photosynthesis and respiration (Kirschbaum, 2000). Such changes may affect the way plants develop (Norby and Luo, 2004) and have the potential to adversely affect metabolic processes in trees (Lavender and Silim, 1987), particularly in periods when trees are undergoing critical physiological processes like bud formation or bud break.

The timing of bud burst is fundamental to tree survival and growth in temperate and boreal regions of the world (Sakai and Larcher, 1987). Precocious bud break in winter or spring can expose developing stem and leaf tissue to frost injury, while delayed bud break may shorten the growing season. In flower or cone buds, these changes can affect the timing and success of pollination and the growing season time available for development of fruits, cones, and seeds.

The three most important factors controlling the phenology of dormancy in temperate and boreal trees are the day length or photoperiod for dormancy induction (Wareing, 1953; Hannerz, 1999), chilling requirement for the transition from endodormancy to ecodormancy (Vegis, 1964), and heat sum requirement to initiate bud break. Generally, a rise in temperature will induce bud opening and flushing of the tree (Cannell and Smith, 1984), and the heat sum required for bud opening, expressed as accumulated degree days above a threshold temperature decreases with increasing duration of chilling (Murray et al., 1989) until the chilling requirement is met.

Precocious bud break and flowering during mild weather in winter is frequently observed in ornamental species planted outside their natural range, and could also occur in natural populations as a result of global warming. Mild weather in winter may also result in loss of frost tolerance, increasing the risk of frost injury in stems and leaves or needles of evergreen species (Strimbeck et al., 1995). Global warming will likely increase the frequency and severity of midwinter mild weather events, especially at higher latitudes, with subsequent risk of frost injury in trees and other plants even within their current natural ranges.

Another possible consequence of global warming is that the chilling requirement might not be fully met and result in delayed bud break (Heide, 1993; Morin et al., 2009). Results of a model of bud break in 22 temperate deciduous trees species, calibrated using extensive data on chilling and heat sum requirements, suggest that bud break in most species may be advanced from a few days to more than 3 weeks under the moderate A2 warming scenario (+1°C). Under the more extreme B2 (+3.2°C) scenario bud opening may be delayed in all or some parts of the current range of some species. More recent analyses suggest that even more extreme warming is possible or even likely. Thus, delayed bud opening and subsequent developmental problems are a real possibility for many boreal and temperate tree and other plant species.

In addition, elevated atmospheric CO_2_ may indirectly affect bud phenology by changing its biochemistry and physiology. For example, variations in starch or phytohormones may alter the dormancy period and growth by shifting phenological patterns such as an earlier flowering or increased duration of vegetative season (Cannell, 1990). In Norway spruce (*Picea abies*), the timing of bud break was associated with a high rate of net photosynthesis followed by decrease in amount of sugar and rise in the starch compounds (Egger et al., 1996). Furthermore, in early spring before bud break, a large amount of carbon is exported out of the needles, while after bud break, starch concentration decreases in tissue and non-structural carbohydrates and hemicelluloses double in quantity.

Sugars and other compatible solutes exert cryoprotective properties and accumulate in conifer cells during frost hardening. Although sucrose is the most common plant sugar, oligosaccharides such as raffinose and stachyose are closely associated with frost tolerance in conifers (e.g., Strimbeck et al., 2007). Major changes in sugar metabolism occur in conifer needles during acclimation and deacclimation (Larcher, 2003; Angelcheva et al., 2014). Cold acclimation generally inhibits growth, however elevated CO_2_ induces a delay in acclimation along with increased risk of frost damage to plants (Loveys et al., 2006).

In evergreen conifers, carbohydrates and photosynthates from the previous year's needles support shoot growth until new needles develop (Hansen and Beck, 1994). At the time of bud break, carbon supply is provided exclusively by metabolic activity of stored reserves, but is considered less important for the development of new shoots (Hansen and Beck, 1990). Old needles in *Picea abies* accumulate large resources of starch prior to bud break, which are used up completely at the time of shoot development (Senser et al., 1975).

Kalberer et al. (2006) specifically point to the need for a better understanding of evolutionary history and ecological significance of the deacclimation phase by addressing signal transduction, gene expression and mechanisms of adaption, including metabolism of proteins and small molecules (metabolites). High-throughput metabolomics platforms and technologies have been introduced in the plants sciences in the past decade. In recent years, gas chromatography/ mass spectrometry (GC/MS)-based metabolite profiling has been successfully applied to dissect molecular mechanisms, regulation of primary metabolism and metabolic networks in different plant genera (Fiehn et al., 2000; Roessner et al., 2000; Lisec et al., 2006), both with respect to cold acclimation (e.g., Cook et al., 2004; Rohloff et al., 2012; Angelcheva et al., 2014) and the deacclimation phase (e.g., Oono et al., 2006; Byun et al., 2014). Early metabolomic reports in conifer species focused on methodology and the establishment of chemometric algorithms for plant selection and phenotyping (Robinson et al., 2007; Zou and Tolstikov, 2008), association genetics (Eckert et al., 2012), and plant regeneration of somatic cultures (Robinson et al., 2009). In more recent studies, questions related to cold acclimation in *Picea sitchensis* (Dauwe et al., 2012) and *Picea obovata* (Shiryaeva et al., 2012; Angelcheva et al., 2014) have been addressed using GC/MS-based metabolite profiling. Only a few reports have approached the deacclimation phase with regard to elevated temperatures, ozone levels (Riikonen et al., 2012), and impact of day length on bud development and bud burst (Lee et al., 2014). Comprehensive studies on regulation of metabolism during bud break and dehardening in conifers is still lacking.

We examined metabolite changes in conifer buds and needles of two evergreen conifers during artificial forcing to measure potential consequences due to early arrival of spring. *Picea abies* L. (Norway spruce) is a boreal species adapted to low temperature ranges that grows throughout northern Europe including Scandinavia and western Russia. *Abies alba* L. (European silver fir), is a temperate species, naturally occurring in central Europe. *Picea abies* is an important commercial species over much of its range, growing up to 50 m height, while *Abies alba* represents one of the largest and most important forest trees in Central Europe (Dobrowolska, 1998). We used GC/MS-based metabolite profiling to examine changes in primarily hydrophilic metabolite pools in bud and needle tissue of both species during bud break and dehardening. We report on changes in levels of carbohydrates, amino acids, Krebs cycle-related compounds and secondary compounds that are more or less consistent between the two species, the needle and bud tissue, or both. Our results provide new insights into potential effects of predicted global warming on phenological characters and biochemical responses in conifers during deacclimation with regard to differences between species and tissue type, and timing of metabolic regulation during bud break.

## Materials and methods

### Location

Plant samples were collected at Ringve Botanical Garden, NTNU, located on the Lade peninsula 3 miles east of downtown Trondheim, Norway (63° 25′, 10° 25′) and a few meters above sea level. Sample trees were all planted right after the establishment of the arboretum in 1973 (Strimbeck et al., 2007). Like most of the coastal regions in Norway, conditions were optimal for plant growth based on the moderation of climate due to the North Atlantic Current, a pole-ward flowing branch of the gulf stream. Based on the Norwegian climate system, local climate is classified as southern boreal and moderately oceanic (Moen, 1999) and, according to a more generalized way of global climate classification, it is described as a temperate oceanic climate.

### Sampling design and incubation

Twigs with buds from species *Picea abies* and *Abies alba* were collected in mid-March 2010, (total of 216 samples), comprising 4 trees per species and 3 samples per tree. In order to score the symptoms of bud break on a weekly basis, individual shoots with needles and terminal buds were established under greenhouse conditions. Shoots were inserted in saturated floral foam blocks (24 shoots per block) in trays with clear plastic tops under quartz halogen lights providing 200 μmol m^−2^ s^−1^ PAR. Samples were kept at continuous 22°C under a 16/8 h day/night cycle for up to 9 weeks. Once a week all remaining samples were scored for bud break and one floral form block replicate was withdrawn. Bud and needle samples were frozen in liquid N_2_ and stored at −80°C for later analysis. For bud sampling, only terminal buds (2, up to 3 buds) were used. Bud and needle samples (shoot 1 to 3) from three single trees (tree no. 1 to 3) were pooled (*n* = 3) to give a total of 108 samples (2 species × 2 tissues × 3 replicate trees × 9 weeks) for metabolite extraction and subsequent GC/MS analysis.

### Scoring of bud break

In the family Pinaceae, the terminal shoots originate from a pre-formed bud. Swelling and bud burst was recorded once a week for the two uppermost buds according to a scale ranging from 0 to 8, in which the 0 stage was referred to dormant and stage 8 to fully differentiated shoots with full-fledged basal needles (Krutzsch, 1973): **0**—Dormant buds; **1**, Buds slightly swollen, needles below buds bent backwards and outwards; **2**, Buds swollen, green to gray green in color, bud scales still closed; **3**, Burst of bud scales, tips of needle emerging; **4**, First elongation of needles to about double bud length; **5**, First spread of needles, buds have now the appearance of a painter's brush; **6**, Elongation of shoot, basal needles not yet spread; **7**, Differentiation of shoot, basal needles spread; **8**, All needles more or less spread, new buds developing.

### GC/MS-based metabolite profiling

Sample extraction for analysis of polar compounds was carried out according to the method described in Sissener et al. (2011) and Uleberg et al. (2012) with some modifications. Pooled samples from needles and buds from *A. alba* and *P. abies* were coarsely crushed under liquid N_2_, and 300 mg of bud and 500 mg of needle tissue were transferred to 15 ml tubes. 2000 μl of pre-cooled 100% methanol with ribitol as internal standard (200 μg/ml) was added to samples and vortexed. After treatment for 1 h at 70°C on a shaking incubator, 1 ml chloroform was added to the tubes, and finally 1 ml H_2_O was added following vortexing. Samples were centrifuged for 10 min at 3000 rpm at 4°C. Aliquots from the clear supernatant of bud (800 μl) and needle extracts (500 μl) were transferred into 1.5 ml round bottomed Eppendorff tubes and further processed according to the protocol described in Sissener et al. (2011) and stored at −20°C prior to GC/MS.

A Varian Star 3400 CX gas chromatograph coupled with a Varian Saturn 3 mass spectrometer was used for all analyses. Sample volumes of 1 μl were injected with a split ratio of 25:1. GC separations were carried out on a HP-5MS capillary column (30m × 0.25mm i.d., film thickness 0.25 μm). Injection temperature was 250°C, and the interface was set to 250°C. The carrier gas was helium at a constant flow rate of 1 ml/min. GC temperature was held isothermically at 70°C for 5 min, ramped from 70°C to 250°C at a rate of 5°C/min, and held at 250°C for 10 min. Temperature was further ramped from 250°C to 275°C at a rate of 25°C/min, and finally held at 275°C for 11 min (analysis time: 63 min). The MS source was adjusted to 220°C and a mass range of *m/z* 70–550 was recorded. All mass spectra were acquired in EI mode.

GC/MS data integration, normalization (total signal) and alignment was carried out using the Metalign software (Lommen, 2009). Based on distinct quantifier ions, detected analytes were assessed quantitatively based on the internal standard ribitol and finally expressed as μg/g FW. Chromatogram visualization and peak identification was carried out using different GC/MS software packages: *Saturn GC/MS Workstation* (Varian Analytical Instruments, Walnut Creek, CA), *AMDIS* software (v. 2.71; National Institute of Standards and Technology NIST, Boulder, CO), and the open source program *OpenChrom* Community Edition (v. 0.9.0) (Wenig and Odermatt, 2010). Tentative compound identification was achieved using the Golm Metabolome Database (GMD) (Hummel et al., 2010) and NIST/EPA/NIH Mass Spectral Library (NIST 05) in combination with an *in-house* retention index library of trimethylsilylated (TMS) metabolites detected in different model species and sample matrices. A total of 169 compounds including 80 metabolites, 56 annotated structures, and 33 unidentified metabolite tags were detected (see Supplementary Table 1 and Supplementary Figure 1).

### Statistical analyses

**Figure 1:**
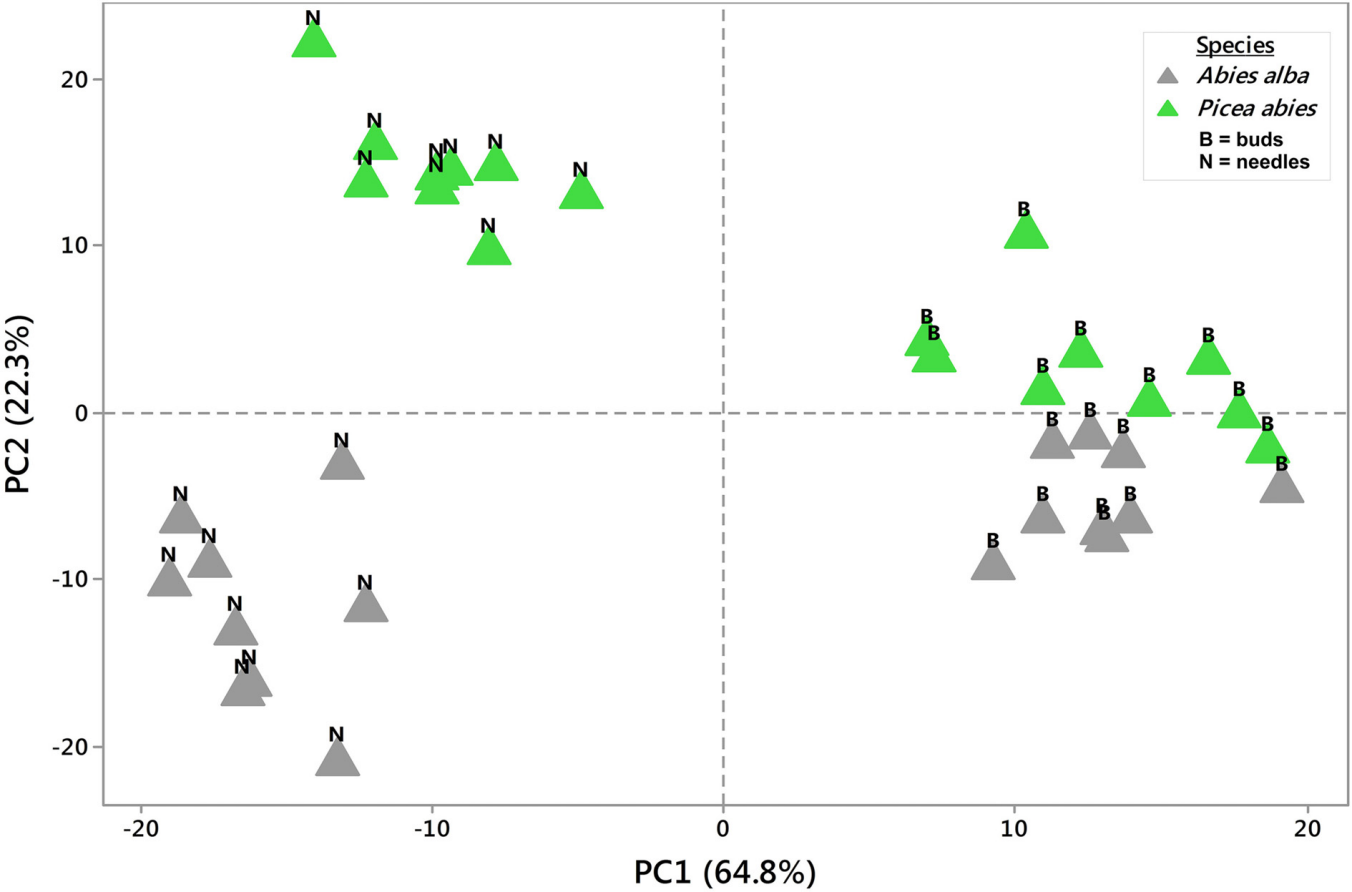
**Principal Component Analysis (PCA)**. 2D PCA was based on PC1 and PC2 from mean concentration values (*n* = 3) of 36 metabolite profiles (813 MS tags each). Samples clearly segregated from each other with regards to tree species or tissue type. PC values were generated from log2(n) values of each single MS tag based on the median from all samples. See also Supplementary Table 1.

**Figure 2:**
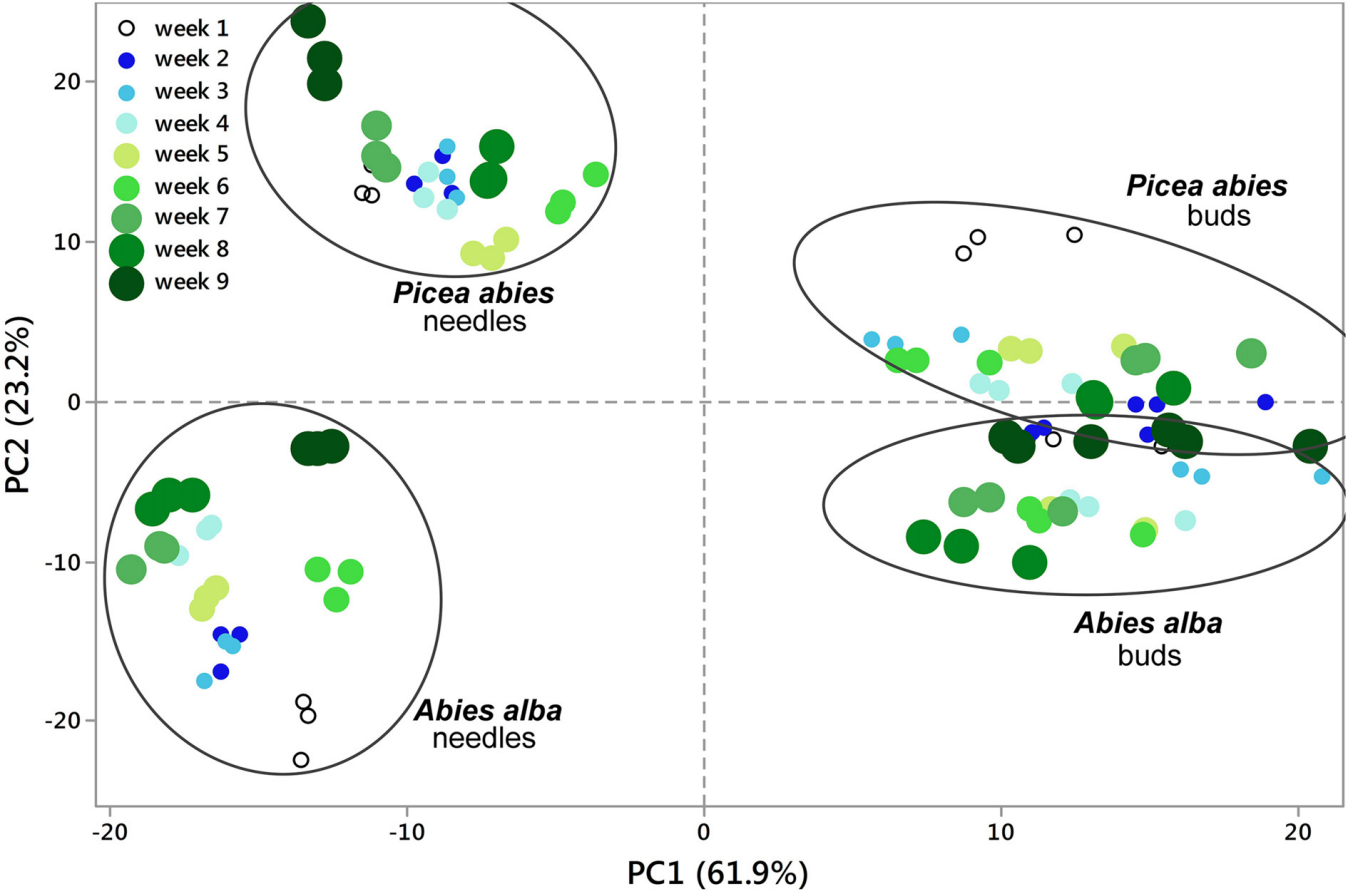
**Principal Component Analysis (PCA)**. 2D PCA was based on PC1 and PC2 from concentration values of all replicates (total of 108 metabolite profiles with 813 MS tags each). Segregation patterns could be shown for samples from week 1 through week 9, being more pronounced in needle tissue (See also Figures 1, 3). PC values were generated from log2(n) values of each single MS tag based on the median from all samples. See also Supplementary Table 1.

**Figure 3:**
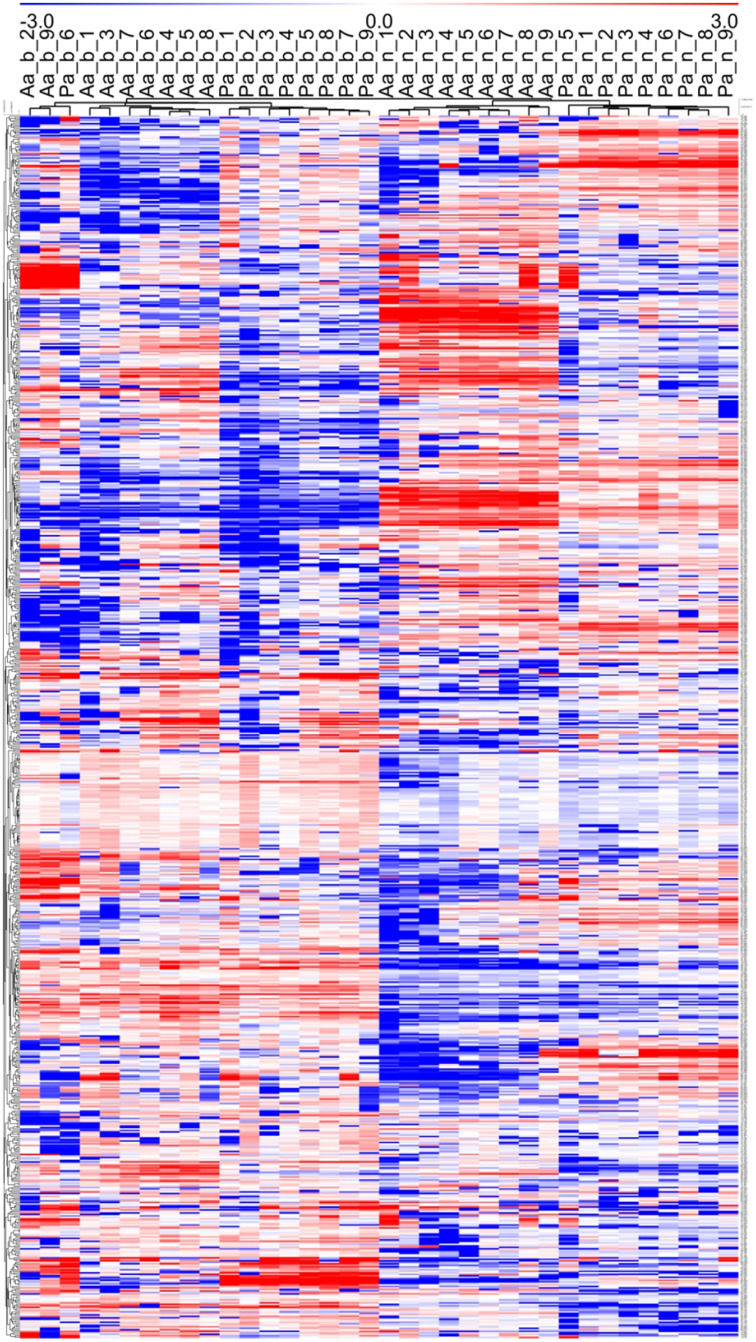
**Hierarchical Cluster Analysis (HCA) based on Pearson correlation calculated from mean concentration values (*n* = 3) of 36 metabolite profiles (813 MS tags each)**. Tree species (Aa, *Abies alba*; Pa, *Picea abies*), tissue type (b, buds; n, needles), and sampling time point (week numbers from 1 to 9) are represented in columns, while distinct MS tags are depicted in rows. The heat map visualization of differences are based on log2(n) ratio amended concentration levels to the median value for each single MS tag. Bluish colors indicate lower metabolite concentrations, while reddish colors show enhanced metabolite levels (see color scale). See also Figures 1, 2, and Supplementary Table 1.

**Figure 4:**
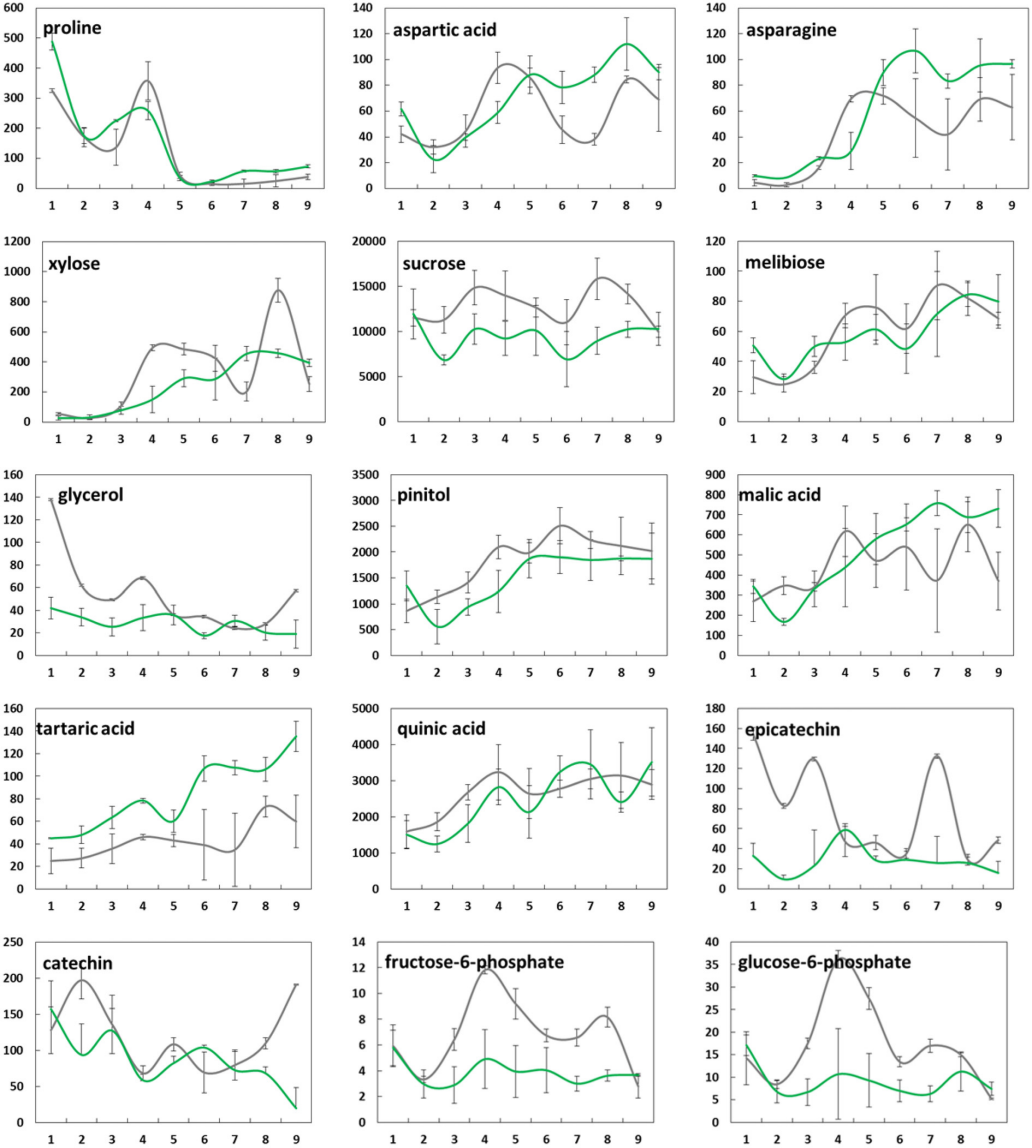
**Changes in mean levels (*n* = 3) of selected metabolites (μg/g FW) in bud tissue from greenhouse-incubated twigs of *Abies alba* (—) and *Picea abies* (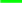) sampled over a 9-weeks period**. Error bars indicate standard deviation. See also Supplementary Figure 1.

**Figure 5:**
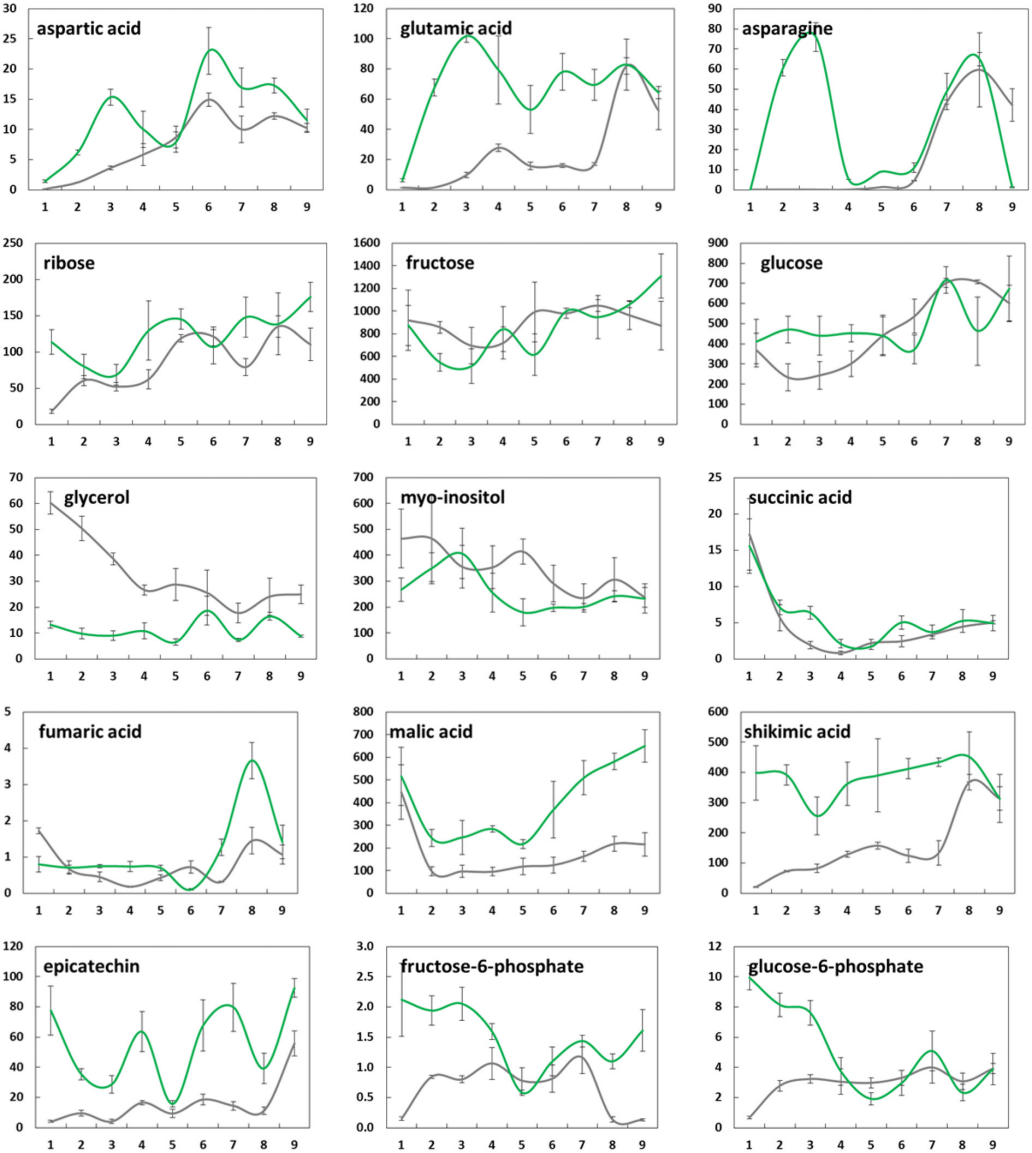
**Changes in mean levels (*n* = 3) of selected metabolites (μg/g FW) in needle tissue from greenhouse-incubated twigs of *Abies alba* (—) and *Picea abies* (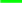) sampled over a 9-weeks period**. Error bars indicate standard deviation. See also Supplementary Figure 1.

**Figure 6:**
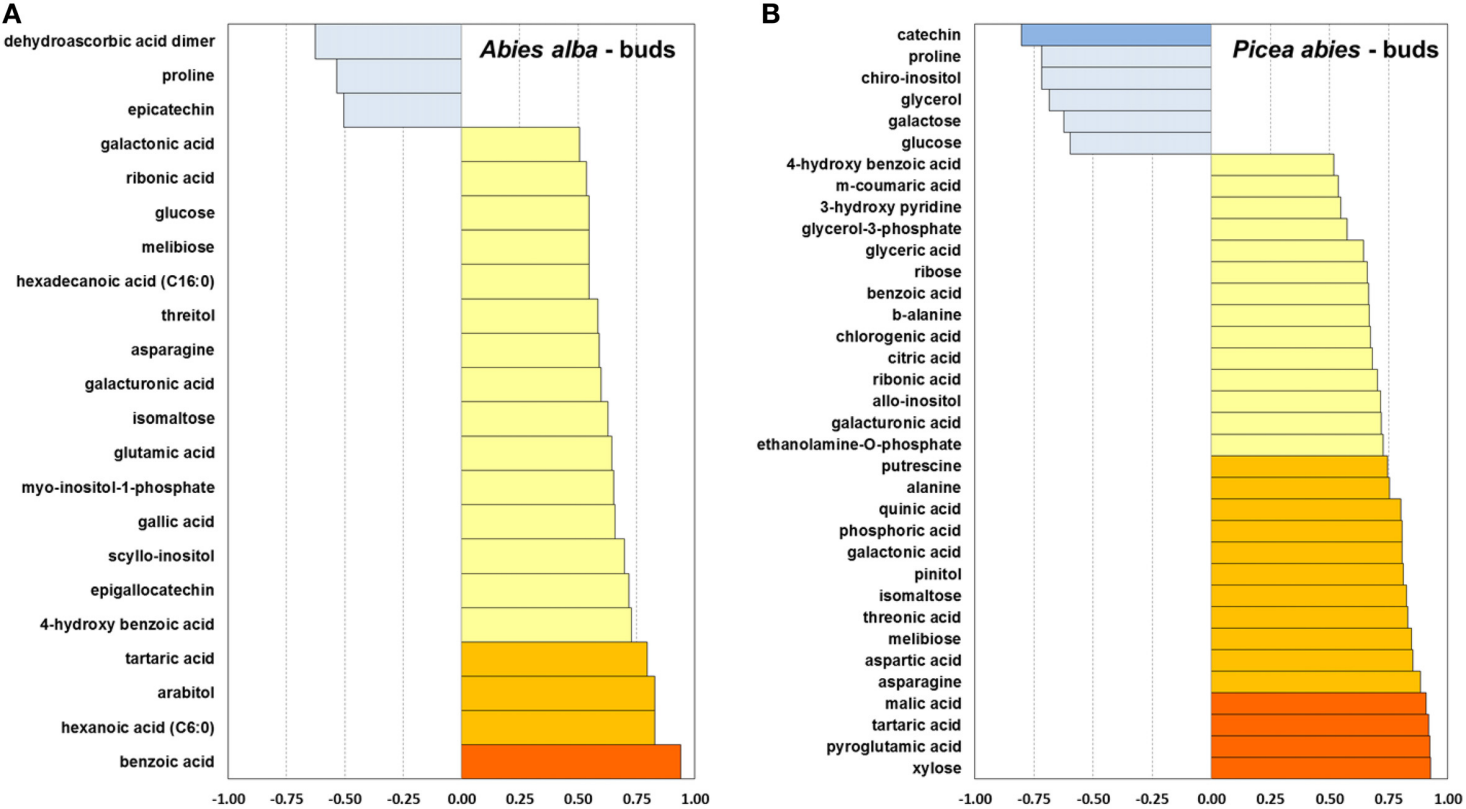
**Pearson correlation between mean bud opening score per week and average metabolite concentration of a total of 80 identified metabolites, calculated for bud tissue of *A. alba* (22 metabolites) (A) and *P. abies* (35 metabolites) (B)**. *r* ≤ −0.75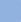; ≤ −0.5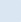; ≥ 0.5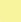; ≥ 0.75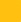; ≥ 0.9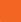.

**Figure 7:**
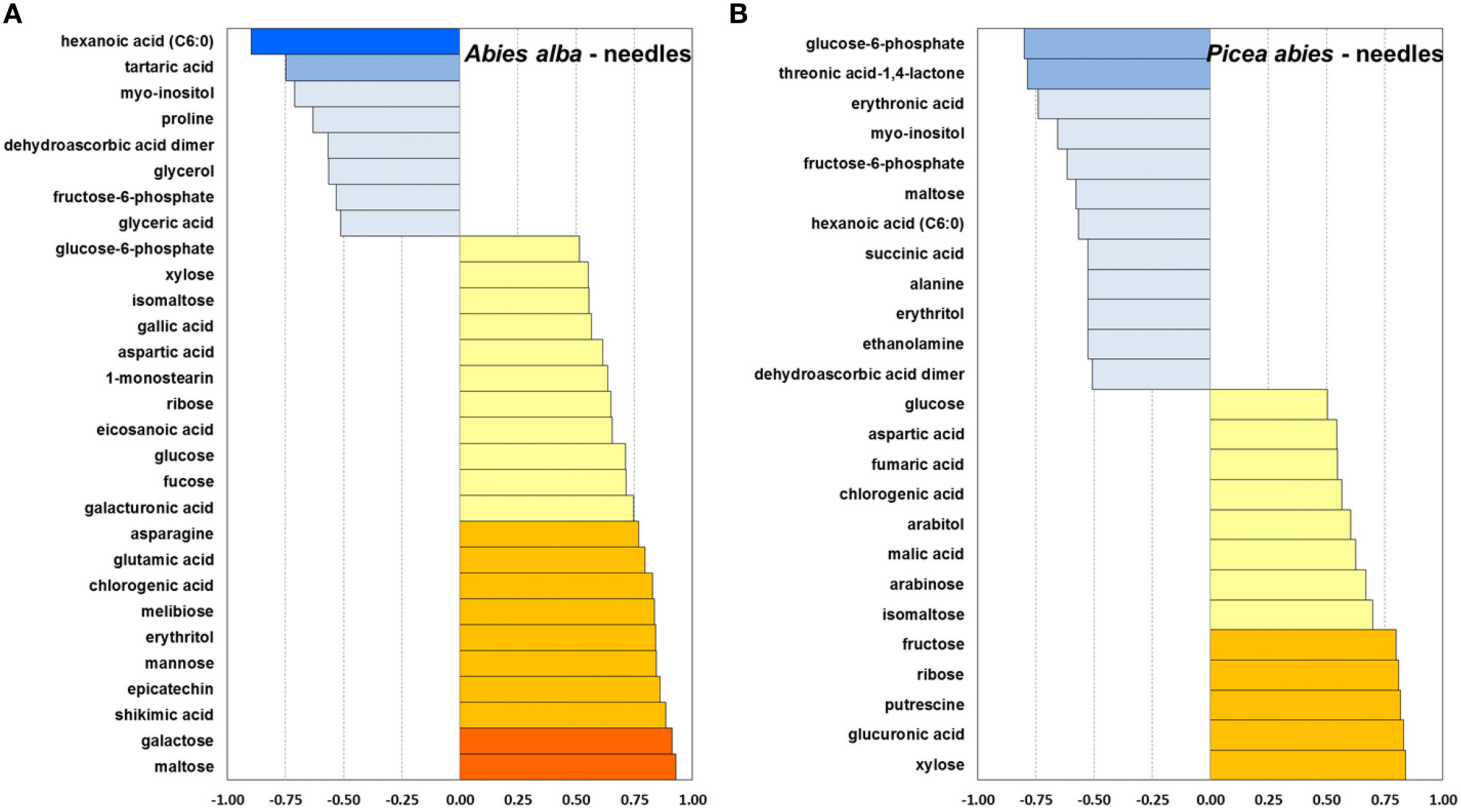
**Pearson correlation between mean bud opening score per week and average metabolite concentration of a total of 80 identified metabolites, calculated for needle tissue of *A. alba* (29 metabolites) (Panel A) and *P. abies* (25 metabolites) (Panel B)**. *r* ≤ −0.9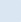; ≤ −0.75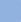; ≤ −0.5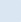; ≥ 0.5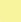; ≥ 0.75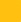; ≥ 0.9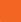.

Aligned data was processed based on a combination of highest MS ion intensity values within each retention time window (scan) and MS abundance (detection in ≥ 70% of all samples). A total of 813 MS tags was filtered out and subjected to *t*-testing and Two-Way ANOVA for evaluating single and combined effects of species (*A. alba* and *P. abies*), tissues (buds and needles), and timepoints (9 weeks). Multivariate analyses were carried out by untargeted principal component analysis (PCA) using Minitab® software (v.17.1.0; Minitab, Ltd., Coventry, UK) (Figures 1, 2), and hierarchical cluster analysis (HCA) with Pearson correlation based on log2(n) transformed data (median base) for single metabolite tags, using MultiExperiment Viewer (MeV) software (v. 4.9.0) (Saeed et al., 2003) (Figure 3). Microsoft® Excel 2010 was used for visualization of changes in mean levels of 50 selected metabolites (μg/g FW) in bud and needle tissue from greenhouse-incubated twigs of *A. alba* and *P. abies* from the 9-weeks study (Figures 4, 5; Supplementary Figure 1). Correlations (Pearson r) between bud opening score and levels of 80 identified metabolites were calculated, highlighting metabolites with correlation coefficients of *r* ≤ −0.5 ≤ 1 or *r* ≥ 0.5 ≥ 1 (Figures 6, 7). Based on the total of 80 known and identified compounds, metabolite distance matrices were calculated and visualized as HCA heat maps for either combined bud or needle metabolites using MeV software (Supplementary Figure 2). Volcano scatter plots were drawn with Microsoft® Excel 2010 based on a total of 80 identified metabolites detected in all samples (*n* = 3) of either buds or needles from greenhouse-incubated twigs of *A. alba* and *P*. *abies*. Log2(fold change) values of metabolite concentration at 9-weeks vs. mean of all time points were calculated and plotted against −log10(p) *p*-values of time points (Supplementary Figure 3).

## Results

### Bud phenology

Greenhouse conditions accelerated bud opening, elongation and maturation of shoots and needles. After week 3, many samples started showing changes at the apical zone of twigs indicated by swelling of the buds. Significant differences between the two species could be shown with respect to earlier bud development and slightly higher bud opening scores for *P. abies* compared to *A. alba* (paired *t*-test; *p* = 0.02824). Average values are included in Supplementary Table 1.

### Metabolic shifts upon forced deacclimation

Using an untargeted approach, 813 MS tags representing potential metabolites were initially selected out of 133,814 MS tags from the aligned data set of 108 analysis samples (Supplementary Table 1). Quality of filtered MS tags was further confirmed based on statistical *t*-testing and Two-Way ANOVA, verifying statistical significance of data for at least one condition related to single factors (species, organ, and tissue) or/and combined effects. Based on these comprehensive metabolite profiles, initial PCA analyses were conducted to describe differences between samples with regard to species and tissue type (Figure 1), and timepoint effects (Figure 2). The two conifer species could be clearly separated from each other (Figure 1). The segregation of samples was even more pronounced with respect to bud and needle tissue, also indicating much stronger separation of needle tissue of *A. alba* and *P. abies* compared to the tight cluster of bud samples. A stronger separation of timepoints (weeks) in needle tissue of *A. abies* could also be observed when including all 108 replicates in PCA analysis (Figure 2). PCA results identified sample groups as indicated by hierarchical clustering (HCA) (Figure 3). Tissue types were clearly separated from each other in two major clusters, each showing divided into sub-clusters related to the conifer species. As already pointed out for PCA of timepoints (Figure 2), samples of needle tissue showed an almost ordered clustering for both *A. alba* and *P. abies* starting from week 1 to 9, also including bud samples of *P. abies*, thus indicating strong timepoint effects in metabolite shifts.

PCA and HCA results are further supported by statistical analyses using *t*-tests and Two-Way ANOVA. Out of the total of 169 assigned compounds (Supplementary Table 1), a higher number of metabolites showing significant differences (*p* ≤ 0.001) could be observed for tissue effects, indicating differential modulation of citric acid cycle, amino acid, amine, polyol, and secondary metabolism. Metabolite levels also differed between species, especially those involed in secondary metabolism such as shikimic acid, the central precursor for the biosynthesis of aromatic amino acids and phenolic structures, e.g., gallic acid, epigallocatechin, and chlorogenic acid, and in carbohydrate metabolism and disaccharides in particular. Time point effects were not so clearly pronounced, but central pathways and corresponding metabolite levels were distinctly affected. Significant *Species × tissue* interactions were found for many metabolites related to carbohydrate and secondary metabolism, while *timepoint × tissue* effects were marked for acid, amino acid, and polyol metabolism.

Changes in the concentrations of selected metabolites over the 9 weeks period are presented in Figure 4 (buds) and Figure 5 (needles). Further results for 50 major metabolites are given in Supplementary Table 1. Both showed substantial metabolic changes by deacclimation week 3. Regardless of the tissue type, several metabolites belonging to the same pathway showed similar levels in bud and needle tissue upon warm temperature treatment, indicating some common metabolic process in both tissue types, including amino acid metabolism (aspartic acid–asparagine, isoleucine–valine, and serine–glycine–threonine), carbohydrate metabolism (sucrose–G6P–F6P), fatty acids (C16:0–C18:0), and Krebs cycle intermediates (succinic acid–fumaric acid–malic acid). These findings were further supported by pathway relationships of a total of 80 identified metabolites in bud or needle tissue of both conifer species, when visualized in correlation heat maps (Supplementary Figure 2). The clustered matrix of metabolite-to-metabolite distances provided a comprehensive view of distinctions and similarities in metabolic regulation and emphasized the interconnection of metabolic modules and single pathways (e.g., fatty acids and carbohydrates). In contrast, several compounds not considered as biosynthetic intermediates but rather endpoint metabolites (e.g., secondary metabolites), and/or known compatible solutes (e.g., proline) demonstrated characteristic accumulation or degradation patterns throughout the greenhouse study.

While the concentration of some metabolites in buds declined (e.g., proline, glycerol, and catechin), many metabolites showed clearly increasing levels associated with bud development, growth, and photosynthesis (e.g., citric and malic acid, pinitol, xylose, ribose, melibiose, aspartic acid, and asparagine) (Figure 4). When comparing species, *A. alba* tended to show an earlier onset of metabolic modulation and greater concentration changes compared to *P. abies*. This was particularly true for many pentoses and disaccharides, phosphates, several secondary metabolites, Krebs cycle acids, and amino acids (pyroglutamic acid and GABA). Species differences were also observed regarding the overall level of high-abundant metabolites based on mean values (Supplementary Table 1 and Supplementary Figure 1), including sugars like xylose, ribose, fructose, and sucrose, alcohols (pinitol and *myo*-inositol), acids (quinic acid), and catechin-related compounds. The inititation of metabolism and major biochemical changes in needles of *A. alba* and *P. abies* seemed to be delayed compared to bud tissue (Figure 5 and Supplementary Figure 1). However, most amino acids showed an obvious response to exposure to warm conditions (e.g., alanine, valine, glutamic acid, and aspartic acid), often showing characteristic transient concentration peaks. Significant declines in levels of succinic, fumaric and malic acid at the onset of greenhouse treatment were followed by an increase toward the end of the 9-weeks. Pentoses and hexoses also increased in needles of both species. Several major metabolites of *P. abies* needles (Supplementary Table 1 and Supplementary Figure 1) showed generally higher concentration levels (mean values), comprising amino acids, selected sugars (e.g., maltose and galactose), acids (quinic acid, shikimic acid, and malic acid), and secondary structures (e.g., chlorogenic acid), and thus emphasized biochemical differences between species. Moreover, metabolic shifts upon forced deacclimation often showed inverse trends as exemplified by disaccharides (maltose, melibiose, sucrose, G6P, and F6P) and secondary metabolism (e.g., shikimic acid and catechin) in *A. alba* and *P. abies*.

### Correlation of metabolism with bud development

In *A. alba* buds several carbohydrates and related metabolites (sugar alcohols, acids, and phosphates) showed a medium to strong positive correlation between the weekly measured concentration and the corresponding bud opening score (Figure 6), while in buds of Norway spruce malic acid, citric acid, pinitol, xylose, and ribose correlated closely with bud opening score. Several secondary compounds showed positive correlations with bud opening score in both spcies, but the flavan-3-ols epicatechin (*A. alba*), and catechin (*P. abies*) correlated negatively. Only glutamic acid and asparagine showed increasing levels in line with bud development in *A. alba*, while 5 amino acids were positively correlated in *P. abies* buds. As a common feature, a clearly negative association was found for proline, whereas biosynthesis of tartaric acid was strongly connected with bud opening in both species.

In comparison to buds, the needles of both species indicated relatively stronger positive correlations of sugar-related metabolites (xylose, ribose, fructose and glucose) with bud opening score (Figure 7). Obvious positive relationships were observed for maltose and G6P in *A. alba*, in contrast to *P. abies*. As already pointed out for bud tissue, a relatively strong interconnection of Krebs cycle metabolites was also found in needles of *P. abies* indicated by positive correlations for malic and fumaric acid, and a negative correlation for succinic acid. Moderate to strong negative correlation of metabolite concentrations with bud score in needle tissue was indicated for the sugar alcohol *myo*-inositol in both species, while aspartic acid and chlorogenic acid were positively connected. Moreover, Volcano plots of metabolite fold changes plotted against *p*-values of time points (weeks) were drawn to reveal those compounds with significant changes in concentration levels at the end of the deacclimation period in week 9 (Supplementary Figure 3). Differential regulation of biosynthesis of sugar acids (galactonic acid, galacturonic acid, and glucuronic acid) and sugars (galactose) further pointed at the modulation of ascorbate and aldarate metabolism in dehardening plant tissue. Results further emphasize characteristic shifts in single pathways, both highlighting single compounds with known or potential osmolyte function such as proline (all tissue types), asparagine (needles), isoleucine and valine (needles of *P. abies*), and putrescine (buds of *A. alba*). The latter compound is converted to 4-aminobutyric acid (GABA) and consecutively links glutamate metabolism and Krebs cycle via the GABA shunt (see Supplementary Figure 1 and Table 1), thus indicating the interconnection of metabolic modules in temperature-induced physiological processes.

## Discussion

### Phenology of bud burst

Global warming has the potential to impact on plant growth and development including phenological events such as bud burst, abscission, senescence, and flowering. These events may be affected by an increase in global temperatures resulting in altered developmental rhythm of forest trees toward early timing of onset and cessation of bud burst (Ceulemans and Mousseau, 1994; Morin et al., 2009). In the present study, bud growth patterns were observed over 9 weeks under forced deacclimation and bud break. While bud opening in detached twigs was incomplete after 9 weeks, it appeared normal through the bud swelling stage (Stage 3) and early stages of bud burst. However, results presented here have to be considered with respect to the experimental set-up (controlled environment under constant temperature and unchanged daylight conditions) and potentially disturbed physiological functioning (source-sink relationships) due to the detachment of shoots. In similar studies using forced deacclimation with long-day photoperiods, plants showed increased growth with flushing of needles (Jach and Ceulemans, 1999) and earlier bud burst (Riikonen et al., 2012). Wlevated temperatures in Scots pine led to earlier dehardening and potential frost damage (Hänninen et al., 1993; Repo et al., 2006),. Depending on the length of warm temperature exposure and conifer species, the degree of frost damage might differ greatly (Gooch et al., 2009). In non-conifer tree species like snow gum (*Eucalyptus pauciflora*), relatively small changes in day temperatures during mild weather in early spring, in combination with enhanced CO_2_-levels, might be sufficient to induce frost damage to leaves (Loveys et al., 2006). Any further rise in temperature would counterbalance vegetative productivity in snow gum, also reducing the benefits of high photosynthetic activity (Lutze et al., 1998).

### Changes in metabolite pools related to bud burst and shoot development

Based on GC/MS profiling and MS fragment assessment, a total of 813 MS tags were detected in all samples, of which 169 assigned metabolites were detected, many of which are known to be involved in various metabolic processes in conifers during deacclimation. Given the polarity of the extraction solvent, the detection of fatty acids was naturally low, i.e., the range of detected and identified compounds covered mostly polar metabolites, generally comprising several and different functional groups attached to the molecule. Another factor regarding limitation of compound detection were the applied GC/MS settings in combination with extraction conditions, leaving out both sterols and trisaccharides. However, the latter are functionally involved in cold acclimation processes and acquired frost tolerance as in the case of e.g., raffinose (Rohloff et al., 2012; Davik et al., 2013), and readily catabolized to disaccharides and hexoses during deacclimation (Strimbeck et al., 2008). Tentative identification of compounds based on MS database search, using an in-house MS library of TMS metabolites and retention indices, resulted in a relatively high number of 80 identified metabolites, 55 compounds with known molecular class, and 34 unknown structures (total of 169 assigned metabolites). However, given the initial selection of 813 MS tags and the filter mode, several potential metabolites might have been missed. Depending on instrument sensitivity (TOF vs. ion trap vs. quadrupole) and settings, metabolite profiling studies in various conifer species using GC/MS reported similar results with a total amount of assigned metabolites ranging from 44 to 115 identified compounds (e.g., Businge et al., 2012 [44 metabolites]; Eckert et al., 2012 [86]; Shiryaeva et al., 2012 [115]; Angelcheva et al., 2014 [68]; Lee et al., 2014 [105]), thus emphasizing the quality of GC/MS detection and the method's applicability to capture changes in metabolite pools.

In our study, we present for the first time an in-depth analysis of metabolic regulation during dehardening and bud opening in conifer bud and needle tissue. The total of 80 identified compounds gives a detailed overview of metabolite patterns and changes during forced deacclimation. The fate and patterns of carbohydrate accumulation and degradation could be physiologically explained, revealing the possible allocation and distribution of various metabolites in needles and buds of *A. alba* and *P. abies*. In general, changes in carbohydrate levels and other solutes are associated with seasonal variations of acclimation and deacclimation in woody species (Cox and Stushnoff, 2001; Strimbeck et al., 2008).

Unfortunately, the present experimental setup limited the possibility of sugar export because twigs detached from the parental plant were sampled and grown in the greenhouse, with no possibility for translocation of photosynthates and metabolites into the roots. Since previous year needles of the young shoots were fully differentiated and capable of photosynthesis, photosynthetic gain was potentially very high in needles. Photosynthates in the newly formed needles and buds are the primary source of carbon for the development of shoots and an overall increase in spring sugar is well documented (Fischer and Höll, 1991). Once needles attain half of their final growth and can readily supply other photosynthetic products, these are translocated to the roots (Ericsson, 1978). However, in our experiment translocation was inhibited due to lack of root system.

Changes in the concentration levels of distinct carbohydrates appeared to contribute more to needles than buds. These changes in sugars, which can act as osmolytes, might enhance water movement toward the developing bud (De Faÿ et al., 2000). In wood of deciduous trees, products of photosynthesis decrease in quantity toward the final stage of bud burst, followed by the flushing of new shoots (Kozlowski and Keller, 1966). However, in conifers the development of new shoots is not dependent on the reserve accumulates from previous year needles, i.e., shoots can easily support themselves with photosynthetic material. By the end of needle development, the rate of photosynthesis increases along with increased mobilization of metabolites in the needles and buds, and similarly, metabolite levels in stems are enhanced up to several fold in the late summer (Hansen and Beck, 1994). Photosynthetic enhancement in relation to temperature is well-documented (Corcuera et al., 2005) which infers that temperature enhances the use of carbon reserve in plants. Thus, the present study demonstrates similar effects of early spring temperature, when buds and needles display clearly demarcated differences in response to temperature, acquired in the greenhouse.

Conifers have evolved several mechanisms to cope with unfavorable climate changes. In *Pinus sylvestris*, low temperature results in a reduction in photosynthetic activity and subsequently lead to a decline in primary growth (Ensminger et al., 2006). In nature, deacclimation starts at a slow rate and gradually reaches its maximum during the onset of spring. After dormancy break and the plants are dehardened, it is impossible to switch back to acclimation phase again. This effect is called passive and slow deacclimation; hence conifers attain photosynthetic activities at very slow rate. In our study under controlled environmental conditions, an increase in common products of photosynthesis was observed at early stages, together with the initiation of metabolism. Once cold acclimated plants are exposed to elevated and “unnaturally” high temperatures, deacclimation starts within 2 or 3 h (Chen and Li, 1980), and hardiness might be lost during one or only few days, as discussed by Kalberer et al. (2006). Direct application of constant elevated temperatures (22°C) under greenhouse conditions resulted in high metabolic activity, i.e., photosynthesis was assumed to be efficient as indicated by a steady increase in sugars after an initial concentration drop (e.g., fructose and glucose) (Supplementary Figure 1), which is also confirmed by positive metabolite correlations (Figures 6, 7). In nature, we rather see the opposite phenomenon with gradually rising temperatures, initiation of cell respiration, and breakdown of storage sugars and osmolytes. Simultaneously, photosynthesis is still suboptimal, not gaining enough assimilates to make sugar levels increase, thus fructose and glucose levels are expected to drop under dehardening of cold-acclimated plant tissue under natural environmental conditions (Strimbeck et al., 2008). In contrast, conifer buds in our study did not show such patterns not least because of the need to establish photosynthetic functions, and due to rapidly metabolized assimilates.

The observed patterns of metabolite degradation could be associated with requirement of components for synthesis of cell wall material and other energy requirements in developing needles (Hansen and Beck, 1990). *A. alba*, which is naturally adapted to warmer climates compared to Norway spruce, also showed an earlier onset of increasing levels in energy-related metabolites such as phosphoric acid and key intermediates in glycolysis/gluconeogenesis like F6P and G6P. These observations are further supported by generally increasing levels of Krebs cycle acids in buds (Supplementary Figure 1), i.e., citric and malic acid in both species, and succinic and fumaric acid (in *P. abies*). The positive correlation of Krebs cycle acids with bud opening score in buds and needles of *P. abies* (Figures 6B, 7B) demonstrated the key role of the Krebs cycle in relation to photosynthesis, central metabolism and energy production.

A comparison of bud and needle metabolism of *A. alba* and *P. abies* using Pearson correlation (data not presented), revealed that changes in concentration levels of highly abundant central metabolites often showed similar trends in both conifer species (Supplementary Figure 1). Important compounds included: glucose (needles: *r* = 0.54), fructose (buds: 0.69) and xylose (buds: 0.60; needles: 0.58), Krebs cycle acids citric acid (buds: 0.75), and malic acid (needles: 0.63), and pathway-related compounds like quinic acid (buds: 0.75) and pinitol (buds: 0.73). In contrast, many metabolites showed inverse modulation patterns between species, including disaccharides, amino acids, and secondary compounds, i.e., both timing and degree of modulation of metabolite pools differed greatly between the Central European species *A. alba* and *P. abies* originating from Northern Europe.

### Modulation of metabolite pools related to accumulated osmolytes

Conversion of starch to soluble sugars and increases in carbohydrate concentration are common in plants during acclimation and adaption to freezing stress (Sakai and Larcher, 1987). Nonetheless, accumulation of compatible solutes with cryoprotectant function is not limited to sugars and their oligomers, but is rather linked with antioxidant activity and modulation of other solutes and polymeric structures as well, such as amino acids and amines, organic acids, polyols, phospholipids, and proteins (Ruelland et al., 2009). It has been shown that accumulation/degradation of sugars during acclimation/dehardening differed between conifers originating in boreal and temperate regions (Strimbeck et al., 2007, 2008). This implies that a direct comparison of distinct metabolite concentrations between the investigated species in our study might be misleading, and one should rather focus on onset and timing of metabolic changes in bud and needle tissue. In the same way as plant cold acclimation is achieved through orchestration of gene expression and underlying gene regulatory and metabolic networks as first shown in the plant model *Arabidopsis thaliana* (e.g., Cook et al., 2004; Kaplan et al., 2004, 2007), deacclimation and potential reacclimation are similarly characterized by coordinate regulatory patterns (Byun et al., 2014). Obviously, the dynamic nature of plant dehardening is not only related to genotypic differences, but also directly determined by environmental conditions during the plant's acclimation phase (Kalberer et al., 2006), generally termed as memory of cold stress (Byun et al., 2014). Assuming equal cold acclimation conditions, which the parental species and genotypes in our study were exposed to during winter, and due to controlled greenhouse conditions shoot samples were grown under, the differential regulation of metabolism has to be seen in the context of individual genotypic variation (standard deviation), but most importantly with respect to species differences between *A. alba* and *P. abies*.

Conifers under warm temperature are not fully able to deacclimate their tissues below a minimum hardiness level until the dormant state is broken, but once completed, any further increase in temperature can directly affect and increase growth of plants, followed by rehydration (Kalberer et al., 2006). Under natural conditions, plants need to be deacclimated slowly in order to prevent any frost injury to the tissues (Saxe et al., 2001). The forced deacclimation initiated rapid degradation and/or modulation of accumulated osmolytes potentially related to frost tolerance. One of the most prominent metabolites known to be involved in cold tolerance mechanisms is the amino acid proline (Cook et al., 2004). As observed in bud tissue of both conifer species, levels of proline declined rapidly from 300 to 500 μg/g FW to 30 to 70 μg/g FW after the 9-weeks warm temperature treatment (Pearson correlation: *r* = 0.88). This is in accordance with recent findings by Lee et al. (2014), who reported a drastic increase of proline levels in Norway spruce shoot tips during the cold acclimation phase. In contrast, we did not find reductions of other osmolytic amino acids such as glutamic acid, asparagine (Lee et al., 2014) and valine (Angelcheva et al., 2014). We found correlations between species in one or both tissues in various amino acids (Supplementary Figure 1): valine (buds: *r* = 0.82; needles: 0.69), serine (buds: 0.62), threonine (buds: 0.55; needles: 0.66), aspartic acid (buds: 0.52; needles: 0.81), and asparagine (buds: 0.76). *P. abies* showed higher concentration levels of these compounds and moreover, amino acid increases in needles of Norway spruce occurred at an earlier time point, also including alanine, proline and pyroglutamic acid, indicating more pronounced responses of nitrogen metabolism in this species. Similar differential responses in needles were also observed for several pentoses and hexoses, despite tight correlation of metabolite levels between species as already discussed, also including disaccharides such as sucrose, maltose, and melibiose. Generally increasing levels of the latter metabolite indicated sustained degradation of (assumed) high raffinose stores (Cox and Stushnoff, 2001; Strimbeck et al., 2008; Lee et al., 2014), and consecutive mobilization of carbohydrate reserves not any longer needed to maintain frost tolerance.

Pools of several small molecules with known or proposed function as osmsoprotectant, were observed to decrease rapidly during forced deacclimation in needle tissue, including glycerol, malic acid and succinic acid. These findings are in accordance with earlier studies, indicating increasing levels of malate and succinate during cold acclimation in conifers (Dauwe et al., 2012; Angelcheva et al., 2014; Lee et al., 2014), which later might be degraded during the dehardening phase. However detected compound levels were comparably low (except for malic acid), thus the metabolites potential role in osmolyte functioning remains unclear. To which extent secondary metabolites such as simple phenolic structures and polyphenols are directly and functionally involved in cold acclimation/deacclimation processes in plants, apart from their antioxidant activity, is still a matter of discussion. Several authors have reported increasing levels of flavonoids in leaf tissue of conifer species during fall/early winter such as catechin (Dauwe et al., 2012). Our results showing initially decreasing levels of catechin and epicatechin (only *A. alba*) in buds, are in line with studies by Pukacki and Kaminìska-Rożek (2013) who monitored the decline of total flavonoids in *P. abies* during deacclimation, and correspond with other reports using metabolite profiling approaches to characterize accumulation patterns of distinct compounds (e.g., catechin) upon cold acclimation in *P. obovata* (Angelcheva et al., 2014), *P. sitchensis* (Dauwe et al., 2012), and *P. abies* (Lee et al., 2014). Furthermore, our data suggested a tight interconnection of different modules in secondary metabolism, including precursors such as shikimic acid and catechins (buds), and epigallocatechin with gallic acid (needles) (Supplementary Figure 2). Recent functional studies, integrated molecular approaches using advanced bioinformatic tools have shed light on the role of secondary metabolism in plant cold tolerance (e.g., Usadel et al., 2008; Doerfler et al., 2013), and hopefully also will address biochemical processes occurring during the deacclimation phase in the near future.

## Conclusion

Climate change and global warming generally lead to increased mean temperatures and risks of warm spells, resulting in earlier onset of growth, bud burst and potential frost damage in tree species of boreal forests. In the present study we investigated bud phenology of shoots in two conifer species, *Abies alba* and *Picea abies*, during forced deacclimation under greenhouse conditions. GC/MS metabolite profiling of bud and needle tissue was used here for the first time in a comprehensive approach to measure changes in metabolite pools during the time course of 9 consecutive weeks. Based on untargeted multivariate analyses (PCA and HCA) and the detection of 169 assigned metabolites comprising 80 identified compounds, we were able to characterize major changes in levels of carbohydrates, amino acids, organic acids, and secondary metabolites. Correlation analyses revealed the interconnection of pathway-related metabolites and interconnection of metabolic modules. Furthermore, based on the correlation of phenological and metabolite data, and metabolite fold change analysis, we were able to pinpoint those metabolites and structural classes, which were significantly connected with bud burst and initiated growth. We could clearly demonstrate a differential regulation of metabolism in buds and needle leaves of both conifer species. Differences between species were strongly pronounced, though levels of several metabolites from different compound classes showed similar modulation patterns such as amino acids, Krebs cycle acids and sugars. However, the onset and degree of changes indicated differences between the conifer species, adapted to either boreal climates (*P. abies*) and *A. alba* originating from temperate zones. In conclusion, the present findings have shed light on the effects of temperature-induced changes in plant metabolism of initially dormant tissue, and will further support discussions about the effect of predicted global warming on frost tolerance, phenological events, and biochemical changes in trees during deacclimation.

### Conflict of interest statement

The authors declare that the research was conducted in the absence of any commercial or financial relationships that could be construed as a potential conflict of interest.

## References

[B1] Angelcheva L. Mishra Y. Antti H. Kjellsen T. D. Funk C. Strimbeck G. R.. (2014). Metabolomic analysis of extreme freezing tolerance in Siberian spruce (*Picea obovata*). New Phytol. 204, 545–555. 10.1111/nph.12950 25139797

[B2] Breymeyer A. I. Berg B. Gower S. T. Johnson D. W. (1996). Carbon budget: temperate coniferous forests, in Global Change: Effects on Coniferous Forests and Grasslands, eds Breymeyer A. I. Hall D. O. Melillo J. M. Ågren G. I. (Chichester: John Wiley & Sons, Inc), 41–68.

[B3] Businge E. Brackmann K. Moritz T. Egertsdotter U. (2012). Metabolite profiling reveals clear metabolic changes during somatic embryo development of Norway spruce (*Picea abies*). Tree Physiol. 32, 232–244. 10.1093/treephys/tpr142 22310018

[B4] Byun Y.-J. Koo M.-Y. Joo H.-J. Ha-Lee Y.-M. Lee D.-H. (2014). Comparative analysis of gene expression under cold acclimation, deacclimation and reacclimation in *Arabidopsis*. Physiol. Plant. 152, 256–274. 10.1111/ppl.12163 24494996

[B5] Cannell M. G. R. (1990). Modelling the phenology of trees. Silva Carelica 15, 11–27.

[B6] Cannell M. G. R. Smith R. I. (1984). Spring frost damage on young *Picea sitchensis* 2. Predicted dates of budburst and probability of frost damage. Forestry 57, 177–195. 10.1093/forestry/57.2.17714967641

[B7] Ceulemans R. Mousseau M. (1994). Tansley review No. 71: effects of elevated atmospheric CO_2_ on woody plants. New Phytol. 127, 425–446 10.1111/j.1469-8137.1994.tb03961.x

[B8] Chen H. H. Li P. H. (1980). Characteristics of cold acclimation and deacclimation in tuber-bearing *Solanum* species. Plant Physiol. 65, 1146–1148. 10.1104/pp.65.6.1146 16661349 PMC440499

[B9] Cook D. Fowler S. Fiehn O. Thomashow M. F. (2004). A prominent role for the CBF cold response pathway in configuring the low-temperature metabolome of *Arabidopsis*. Proc. Natl. Acad. Sci. U.S.A. 101, 15243–15248. 10.1073/pnas.0406069101 15383661 PMC524070

[B10] Corcuera L. Morales F. Abadía A. Gil-Pelegrín E. (2005). Seasonal changes in photosynthesis and photoprotection in a *Quercus ilex* subsp. ballota woodland located in its upper altitudinal extreme in the Iberian Peninsula. Tree Physiol. 25, 599–608. 10.1093/treephys/25.5.599 15741152

[B11] Cox S. E. Stushnoff C. (2001). Temperature-related shifts in soluble carbohydrate content during dormancy and cold acclimation in *Populus tremuloides*. Can. J. Forest Res. 31, 730–737 10.1139/x00-206

[B12] Dauwe R. Holliday J. A. Aitken S. N. Mansfield S. D. (2012). Metabolic dynamics during autumn cold acclimation within and among populations of Sitka spruce (*Picea sitchensis*). New Phytol. 194, 192–205. 10.1111/j.1469-8137.2011.04027.x 22248127

[B13] Davik J. From B. Koehler G. Torp T. Rohloff J. Eidem P.. (2013). Dehydrin, alcohol dehydrogenase, and central metabolite levels are associated with cold tolerance in diploid strawberry (*Fragaria* spp.). Planta 237, 265–277. 10.1007/s00425-012-1771-2 23014928

[B14] De Faÿ E. Vacher V. nad Humbert F. (2000). Water-related phenomena in winter buds and twigs of *Picea abies* L. (Karst.) until bud-burst: a biological, histological and NMR study. Ann. Bot. 86, 1097–1107 10.1006/anbo.2000.1276

[B15] Dixon R. K. Solomon A. M. Brown S. Houghton R. A. Trexier M. C. Wisniewski J. (1994). Carbon pools and flux of global forest ecosystems. Science 265, 185–190. 10.1126/science.263.5144.185 17839174

[B16] Dobrowolska D. (1998). Structure of silver fir (*Abies alba* Mill.) natural regeneration in the ‘Jata’ reserve in Poland. Forest Ecol. Manag. 110, 237–247 10.1016/S0378-1127(98)00286-2

[B17] Doerfler H. Lyon D. Nägele T. Sun X. Fragner L. Hadacek F.. (2013). Granger causality in integrated GC-MS and LC-MS metabolomics data reveals the interface of primary and secondary metabolism. Metabolomics 9, 564–574. 10.1007/s11306-012-0470-0 23678342 PMC3651536

[B18] Eckert A. J. Wegrzyn J. L. Cumbie W. P. Goldfarb B. Huber D. A. Tolstikov V.. (2012). Association genetics of the loblolly pine (*Pinus taeda*, Pinaceae) metabolome. New Phytol. 193, 890–902. 10.1111/j.1469-8137.2011.03976.x 22129444

[B19] Egger B. Einig W. Sclereth A. Wallenda T. Magel E. Loewe A. (1996). Carbohydrate metabolism in one- and two-year-old spruce needles, and stem carbohydrates from three months before until three months after bud break. Physiol. Plant. 96, 91–100 10.1111/j.1399-3054.1996.tb00188.x

[B20] Ensminger I. Busch F. Huner N. P. A. (2006). Photostasis and cold acclimation: sensing low temperature through photosynthesis. Physiol. Plant. 126, 28–44. 10.1111/j.1399-3054.2006.00627.x23230444

[B21] Ericsson A. (1978). Seasonal changes in translocation of14C from different age-classes of needles on 20-year-old Scots pine trees (*Pinus silvestris*). Physiol. Plant. 43, 351–358 10.1111/j.1399-3054.1978.tb01593.x

[B22] Fiehn O. Kopka J. Trethewey R. N. Willmitzer L. (2000). Identification of uncommon plant metabolites based on calculation of elemental compositions using gas chromatography and quadrupole mass spectrometry. Anal. Chem. 72, 3573–3580. 10.1021/ac991142i 10952545

[B23] Fischer C. Höll W. (1991). Food reserves of Scots pine (*Pinus sylvestris* L.). I. Seasonal changes in the carbohydrate and fat reserves of pine needles. Trees 5, 187–195 10.1007/BF00227524

[B24] Gooch N. J. Nzokou P. Cregg B. M. (2009). Effect of indoor exposure on the cold hardiness and physiology of containerized christmas trees. Horttechnology 19, 72–77.

[B25] Hannerz M. (1999). Evaluation of temperature models for predicting bud burst in Norway spruce. Can. J. Forest Res. 29, 9–19 10.1139/x98-175

[B26] Hänninen H. Kellomaki S. Laitinen K. Pajari B. Repo T. (1993). Effect of increased winter temperature on the onset of height growth of Scots pine: a field test of a phenological model. Silva Fenn. 4, 251–257 10.14214/sf.a15679

[B27] Hansen J. Beck E. (1990). The fate and path of assimilation products in the stem of 8-year-old Scots pine (*Pinus sylvestris*) trees. Trees 4, 16–21 10.1007/BF00226235

[B28] Hansen J. Beck E. (1994). Seasonal changes in the utilization and turnover of assimilation products in 8-year-old Scots pine (*Pinus sylvestris* L.) trees. Trees 8, 172–182 10.1007/BF00196844

[B29] Heide O. M. (1993). Daylength and thermal time responses of budburst during dormancy release in some northern deciduous trees. Physiol. Plant. 88, 531–540 10.1111/j.1399-3054.1993.tb01368.x 28741760

[B30] Hummel J. Strehmel N. Selbig J. Walther D. Kopka J. (2010). Decision tree supported substructure prediction of metabolites from GC-MS profiles. Metabolomics 6, 322–333. 10.1007/s11306-010-0198-7 20526350 PMC2874469

[B31] IPCC. (2007). Climate change 2007: the physical science basis, in Contribution of Working Group I to the Fourth Assessment Report of the Intergovernmental Panel on Climate Change (New York, NY: Cambridge University Press).

[B32] Jach M. E. Ceulemans R. (1999). Effects of elevated atmospheric CO_2_ on phenology, growth and crown structure of Scots pine (*Pinus sylvestris*) seedlings after two years of exposure in the field. Tree Physiol. 19, 289–300. 10.1093/treephys/19.4-5.289 12651572

[B33] Kalberer S. R. Wisniewski M. Arora R. (2006). Deacclimation and reacclimation of cold-hardy plants: current understanding and emerging concepts. Plant Sci. 171, 3–16 10.1016/j.plantsci.2006.02.013

[B34] Kaplan F. Kopka J. Dale W. H. Zhao W. Schiller K. C. Gatzke N.. (2004). Exploring the temperature-stress metabolome of *Arabidopsis*. Plant Physiol. 136, 4159–4168. 10.1104/pp.104.052142 15557093 PMC535846

[B35] Kaplan F. Kopka J. Sung D. Y. Zhao W. Popp M. Porat R.. (2007). Transcript and metabolite profiling during cold acclimation of *Arabidopsis* reveals an intricate relationship of cold-regulated gene expression with modifications in metabolite content. Plant J. 50, 967–981. 10.1111/j.1365-313X.2007.03100.x 17461790

[B36] Kirschbaum M. U. F. (2000). Forest growth and species distribution in a changing climate. Tree Physiol. 20, 309–322. 10.1093/treephys/20.5-6.309 12651447

[B37] Kozlowski T. T. Keller T. (1966). Food relations in woody plants. Bot. Rev. 32, 293–382 10.1007/BF02858663

[B38] Krutzsch P. (1973). Norway Spruce Development of Buds. Internal Report, IUFRO S2.02.11. Vienna: International Union of Forest Research Organisation.

[B39] Larcher W. (2003). Physiological Plant Ecology - Ecophysiology and Stress Physiology of Functional Groups. Berlin; New York: Springer 10.1007/978-3-662-05214-3

[B40] Lavender D. P. Silim S. N. (1987). The role of plant growth regulators in dormancy in forest trees. Plant Growth Regul. 6, 171–191 10.1007/BF00043954

[B41] Lee Y. K. Alexander D. Wulff J. Olsen J. E. (2014). Changes in metabolite profiles in Norway spruce shoot tips during short-day induced winter bud development and long-day induced bud flush. Metabolomics 10, 842–858. 10.1007/s11306-014-0646-x21118421

[B42] Lisec J. N. Schauer N. Kopka J. Willmitzer L. Fernie A. R. (2006). Gas chromatography mass spectrometry-based metabolite profiling in plants. Nat. Protoc. 1, 387–396. 10.1038/nprot.2006.59 17406261

[B43] Lommen A. (2009). MetAlign: an interface-driven, versatile metabolomics tool for hyphenated full-scan MS data pre-processing. Anal. Chem. 81, 3079–3086. 10.1021/ac900036d 19301908

[B44] Loveys B. R. Egerton J. J. G. Ball M. C. (2006). Higher daytime leaf temperatures contribute to lower freeze tolerance under elevated CO_2_. Plant Cell Environ. 29, 1077–1086. 10.1111/j.1365-3040.2005.01482.x 17080934

[B45] Lutze J. L. Roden J. S. Holly C. J. Wolfe J. Egerton J. J. G. Ball M. C. (1998). Elevated atmospheric [CO_2_] promotes frost damage in evergreen tree seedlings. Plant Cell Environ. 21, 631–635 10.1046/j.1365-3040.1998.00296.x

[B46] Moen A. (1999). National Atlas Norway: Vegetation. Hønefoss: Norwegian Mapping Authority.

[B47] Morin X. Lechowicz M. J. Augspurger C. O'Keefe J. Viner D. Chuine I. (2009). Leaf phenology in 22 North American tree species during the 21st century. Glob. Change Biol. 15, 961–975 10.1111/j.1365-2486.2008.01735.x

[B48] Murray M. B. Cannell M. G. R. Smith R. I. (1989). Date of budburst of fifteen tree species in Britain following climatic warming. J. Appl. Ecol. 26, 693–700 10.2307/2404093

[B49] Norby R. J. Luo Y. (2004). Evaluating ecosystem responses to rising atmospheric CO_2_ and global warming in a multi-factor world. New Phytol. 162, 281–293 10.1111/j.1469-8137.2004.01047.x

[B50] Oono Y. Seki M. Satou M. Iida K. Akiyama K. Sakurai T.. (2006). Monitoring expression profiles of Arabidopsis genes during cold acclimation and deacclimation using DNA microarrays. Funct. Integr. Genomics 6, 212–234. 10.1007/s10142-005-0014-z 16463051

[B51] Pukacki P. M. Kamińska-Rożek E. (2013). Reactive species, antioxidants and cold tolerance during deacclimation of *Picea abies* populations. Acta Physiol. Plant. 35, 129–138 10.1007/s11738-012-1055-2

[B52] Repo T. Hänninen H. Kellomäki S. (2006). The effects of long-term elevation of air temperature and CO on the frost hardiness of Scots pine. Plant Cell Environ. 19, 209–216 10.1111/j.1365-3040.1996.tb00242.x

[B53] Riikonen J. Kontunen-Soppela S. Ossipov V. Tervahauta A. Tuomainen M. Oksanen E.. (2012). Needle metabolome, freezing tolerance and gas exchange in Norway spruce seedlings exposed to elevated temperature and ozone concentration. Tree Physiol. 32, 1102–1112. 10.1093/treephys/tps072 22935538

[B54] Robinson A. R. Dauwe R. Ukrainetz N. K. Cullis I. F. White R. Mansfield S. D. (2009). Predicting the regenerative capacity of conifer somatic embryogenic cultures by metabolomics. Plant Biotechnol. J. 7, 952–963. 10.1111/j.1467-7652.2009.00456.x 19906246

[B55] Robinson A. R. Ukrainetz N. K. Kang K.-Y. Mansfield S. D. (2007). Metabolite profiling of Douglas-fir (*Pseudotsuga menziesii*) field trials reveals strong environmental and weak genetic variation. New Phytol. 174, 762–773. 10.1111/j.1469-8137.2007.02046.x 17504460

[B56] Roessner U. Wagner C. Kopka J. Trethewey N. Willmitzer L. (2000). Technical advance: simultaneous analysis of metabolites in potato tuber by gas chromatography-mass spectrometry. Plant J. 23, 131–142. 10.1046/j.1365-313x.2000.00774.x 10929108

[B57] Rohloff J. Kopka J. Erban A. Winge P. Wilson R. C. Bones A. M.. (2012). Metabolite profiling reveals novel multi-level cold responses in the model *Fragaria vesca* (woodland strawberry). Phytochemistry 77, 99–109. 10.1016/j.phytochem.2012.01.024 22370221

[B58] Ruelland E. Vaultier M.-N. Zachowski A. Hurry V. (2009). Cold signalling and cold acclimation in plants. Adv. Bot. Res. 49, 35–150 10.1016/S0065-2296(08)00602-2

[B59] Saeed A. I. Sharov V. White J. Li J. Liang W. Bhagabati N.. (2003). TM4: a free, open-source system for microarray data management and analysis. BioTechniques 34, 374-378. 12613259 10.2144/03342mt01

[B60] Sakai A. Larcher W. (1987). Frost Survival of Plants: Response and Adaptation to Freezing Stress. Berlin; Heidelberg: Springer Verlag 10.1007/978-3-642-71745-1

[B61] Saxe H. Cannell M. G. R. Johnsen Ø. Ryan M. G. Vourlitis G. (2001). Tree and forest functioning in response to global warming. New Phytol. 149, 369–399 10.1046/j.1469-8137.2001.00057.x 33873342

[B62] Senser M. Schötz F. Beck E. (1975). Seasonal changes in the structure and function of spruce chloroplasts. Planta 126, 1–10. 10.1007/BF00389354 24430082

[B63] Shiryaeva L. Antti H. Schröder W. P. Strimbeck R. Shiriaev A. S. (2012). Pair-wise multicomparison and OPLS analyses of cold-acclimation phases in Siberian spruce. Metabolomics 8, 123–130. 10.1007/s11306-011-0304-5 22593724 PMC3337411

[B64] Sissener N. H. Hemre G.-I. Lall S. P. Sagstad A. Petersen K. Williams J.. (2011). Are apparent negative effects of feeding genetically modified MON810 maize to Atlantic salmon, *Salmo salar* caused by confounding factors? Br. J. Nutr. 106, 42–56. 10.1017/S0007114510005726 21418706

[B65] Strimbeck G. R. Kjellsen T. D. Schaberg P. G. Murakami P. F. (2008). Dynamics of low-temperature acclimation in temperate and boreal conifer foliage in a mild winter climate. Tree Physiol. 28, 1365–1374. 10.1093/treephys/28.9.1365 18595849

[B66] Strimbeck G. R. Kjellsen T. Schaberg P. G. Murakami P. F. (2007). Cold in the common garden: comparative low-temperature tolerance of boreal and temperate conifer foliage. Trees 21, 557–567 10.1007/s00468-007-0151-1

[B67] Strimbeck G. R. Schaberg P. G. Dehayes D. H. Shane J. B. Hawley G. J. (1995). Midwinter dehardening of montane red spruce during a natural thaw. Can. J. Forest Res. 25, 2040–2044 10.1139/x95-221

[B68] Uleberg E. Rohloff J. Jaakola L. Trôst K. Junttila O. Häggman H.. (2012). Effects of temperature and photoperiod on yield and chemical composition of Northern and Southern clones of bilberry (*Vaccinium myrtillus* L.). J. Agric. Food Chem. 60, 10406–10414. 10.1021/jf302924m 23033879

[B69] Usadel B. Bläsing O. E. Gibon Y. Poree F. Höhne M. Günter M.. (2008). Multilevel genomic analysis of the response of transcripts, enzyme activities and metabolites in Arabidopsis rosettes to a progressive decrease of temperature in the non-freezing range. Plant Cell Environ. 31, 518–547. 10.1111/j.1365-3040.2007.01763.x 18088337

[B70] Vegis A. (1964). Dormancy in higher plants. Ann. Rev. Plant Physiol. 15, 185–224 10.1146/annurev.pp.15.060164.001153

[B71] Wareing P. F. (1953). Growth studies in woody species. V. Photoperiodism in dormant buds of *Fagus sylvatica*. Physiol. Plant 6, 692–706 10.1111/j.1399-3054.1953.tb08442.x

[B72] Wenig P. Odermatt J. (2010). OpenChrom: a cross-platform open source software for the mass spectrometric analysis of chromatographic data. BMC Bioinformatics 11:405. 10.1186/1471-2105-11-405 20673335 PMC2920884

[B73] Zou W. Tolstikov V. V. (2008). Probing genetic algorithms for feature selection in comprehensive metabolic profiling approach. Rapid Commun. Mass Spectrom. 22, 1312–1324. 10.1002/rcm.3507 18383216

