# Supplementary material for: Metabolite changes in conifer buds and needles during forced bud break in Norway spruce (Picea abies) and European silver fir (Abies alba)
Source: Front Plant Sci. 2014 Dec 11;5:706. doi: 10.3389/fpls.2014.00706 (PMC4263092; doi:10.3389/fpls.2014.00706)

Supplementary Figure 1. Changes in mean levels of a total of 50 metabolites (µg/g FW) in bud and needle tissue from greenhouse-incubated twigs of *Abies alba* (—) and *Picea abies* (—) sampled over a 9-weeks period. Error bars indicate standard deviation.

Supplementary Figure 1 – Amino Acids (1)

Buds **Needles**

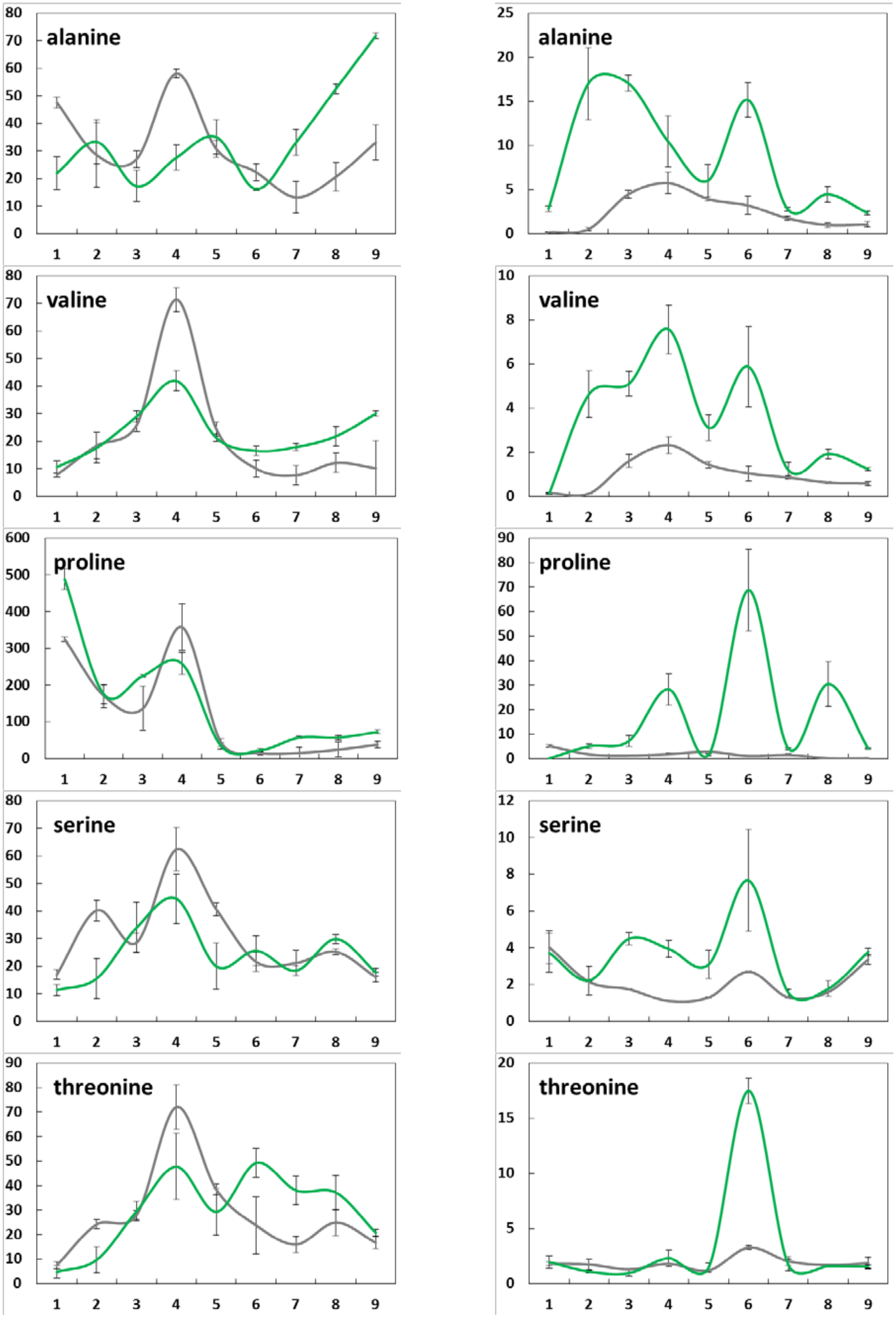

Supplementary Figure 1 – Amino Acids (2)

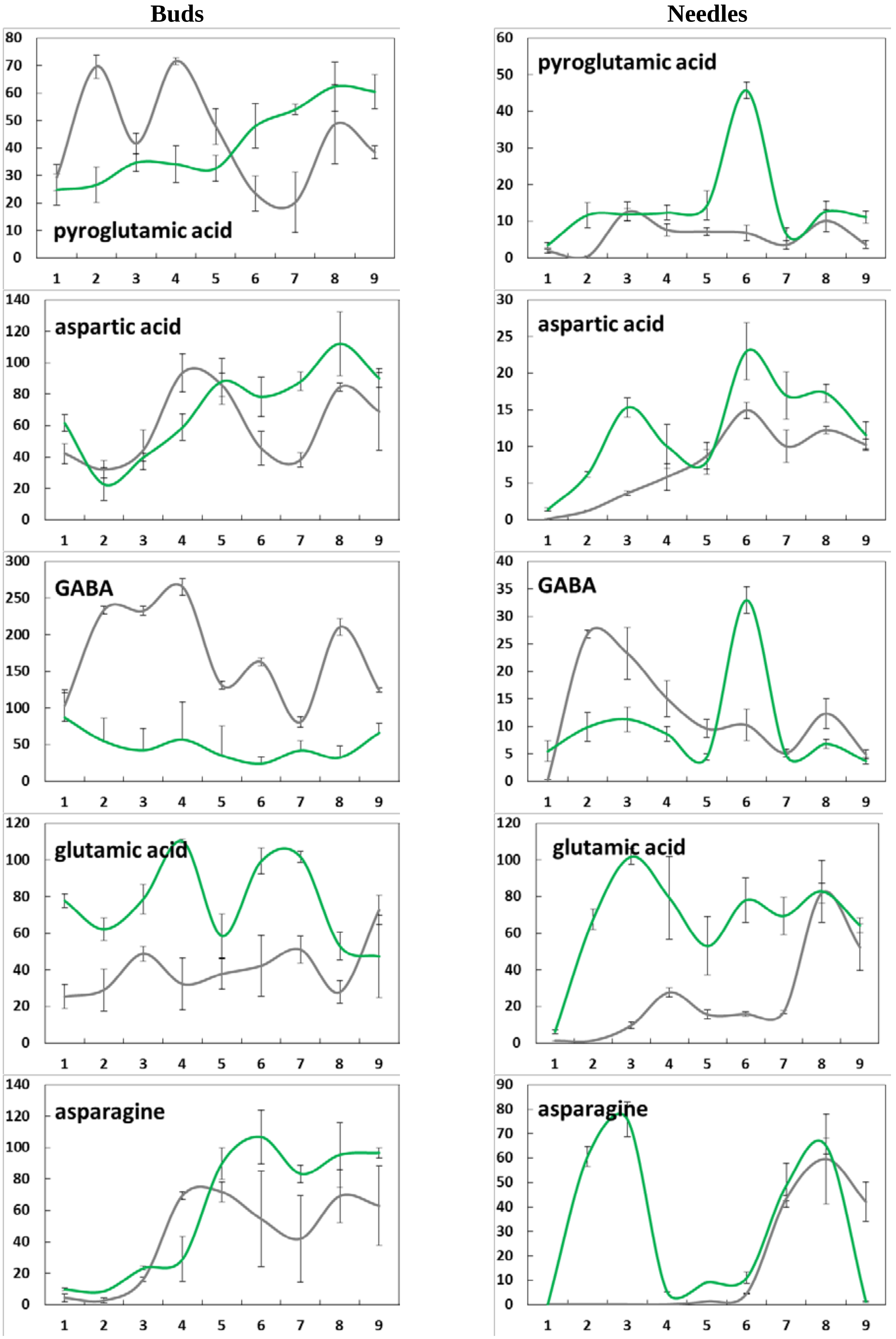

Supplementary Figure 1 – Carbohydrates (1)

Buds | **Needles**

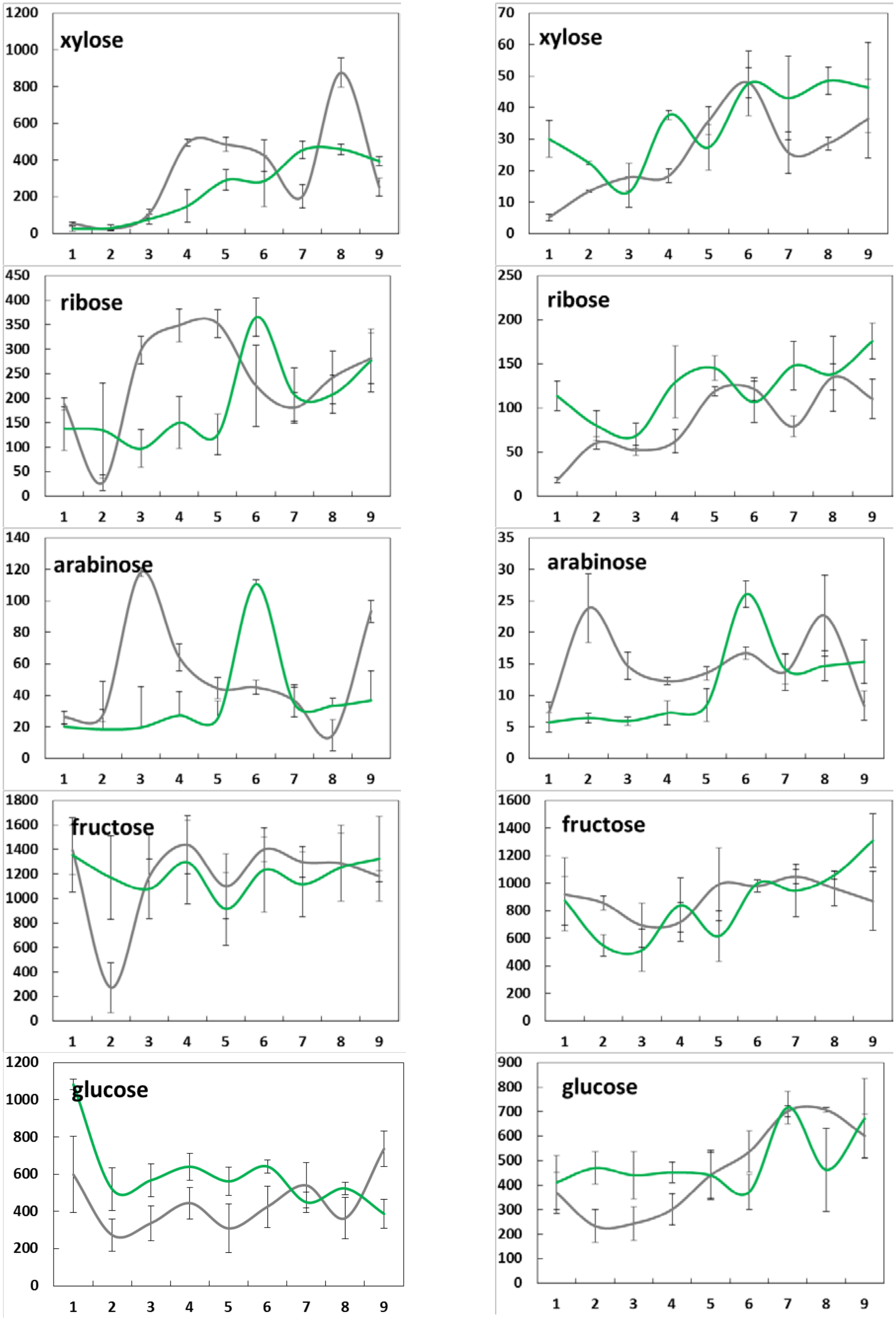

Supplementary Figure 1 – Carbohydrates (2)

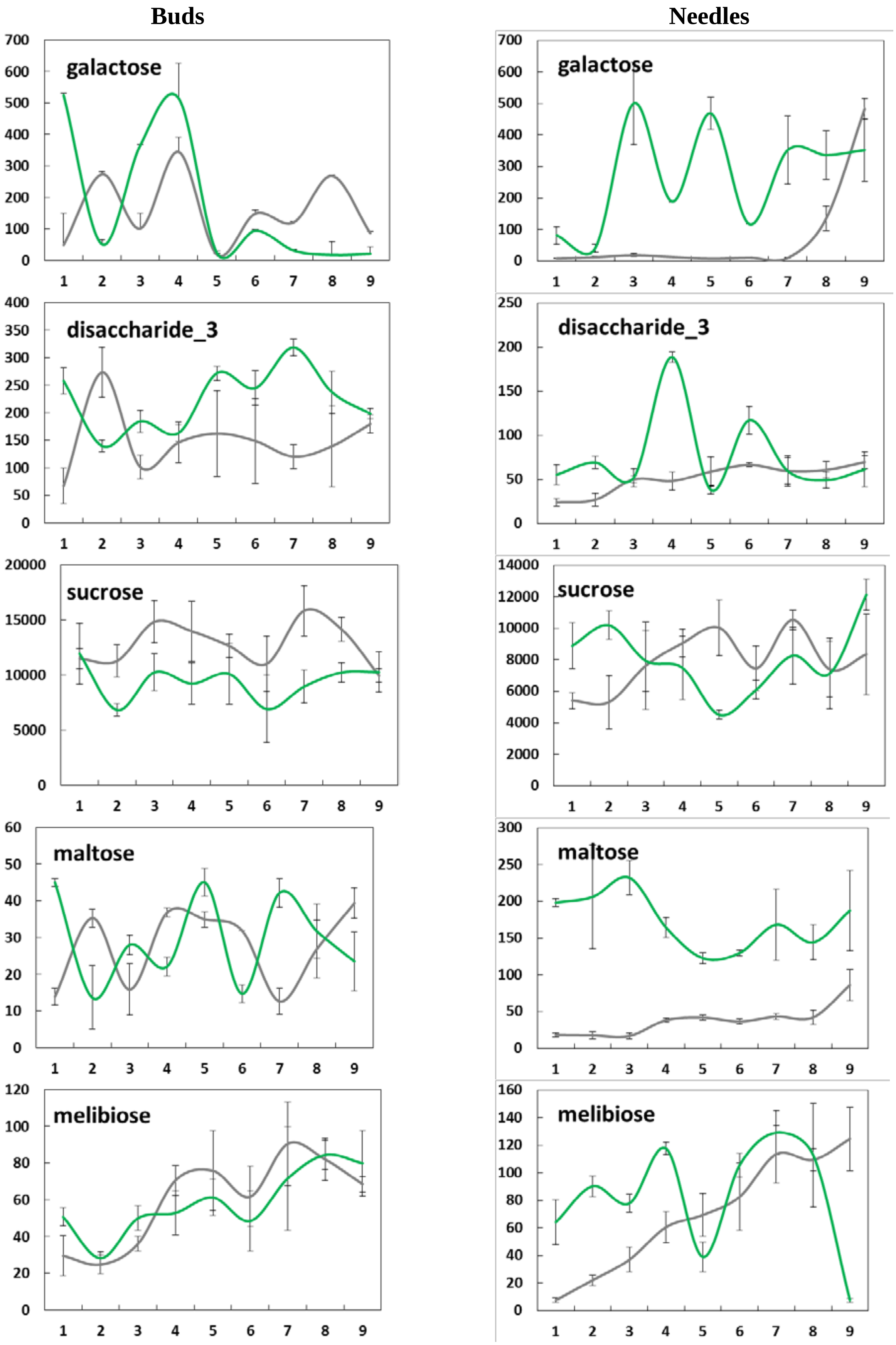

Supplementary Figure 1 – Secondary Metabolites (1)

Buds **Needles**

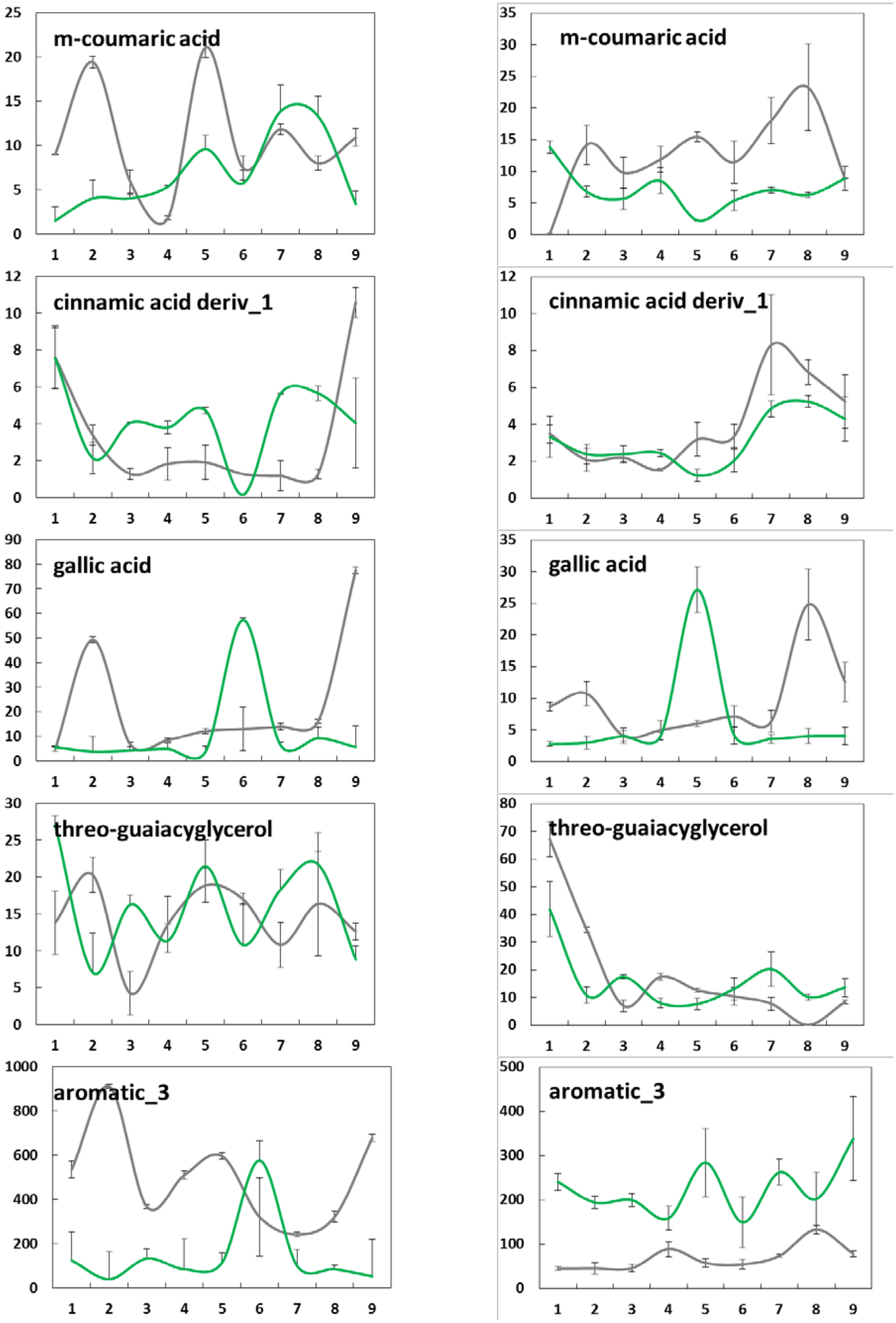

Supplementary Figure 1 – Secondary Metabolites (2)

Buds **Needles**

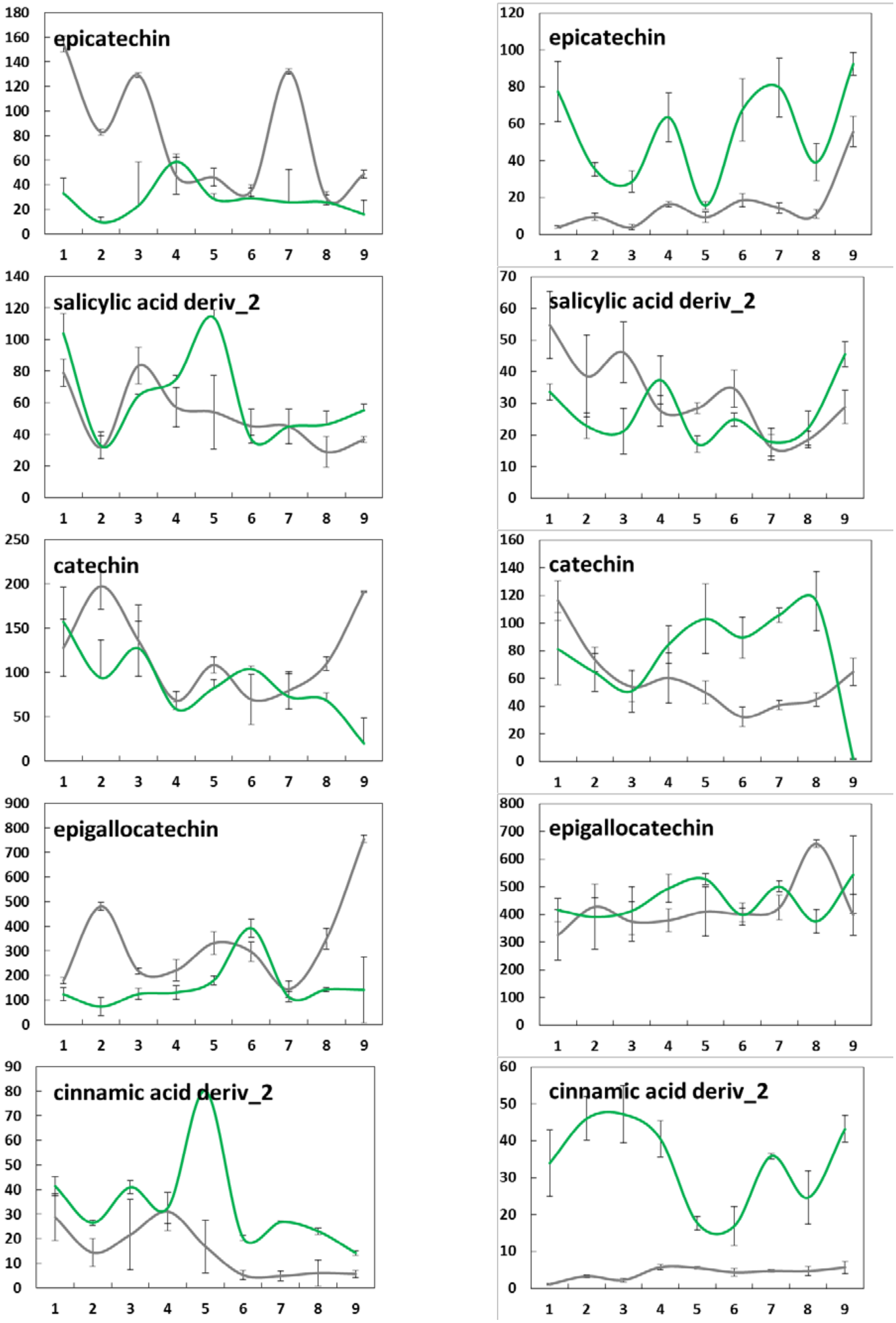

Supplementary Figure 1 – Fatty Acids and Phosphates (1)

Buds | **Needles**

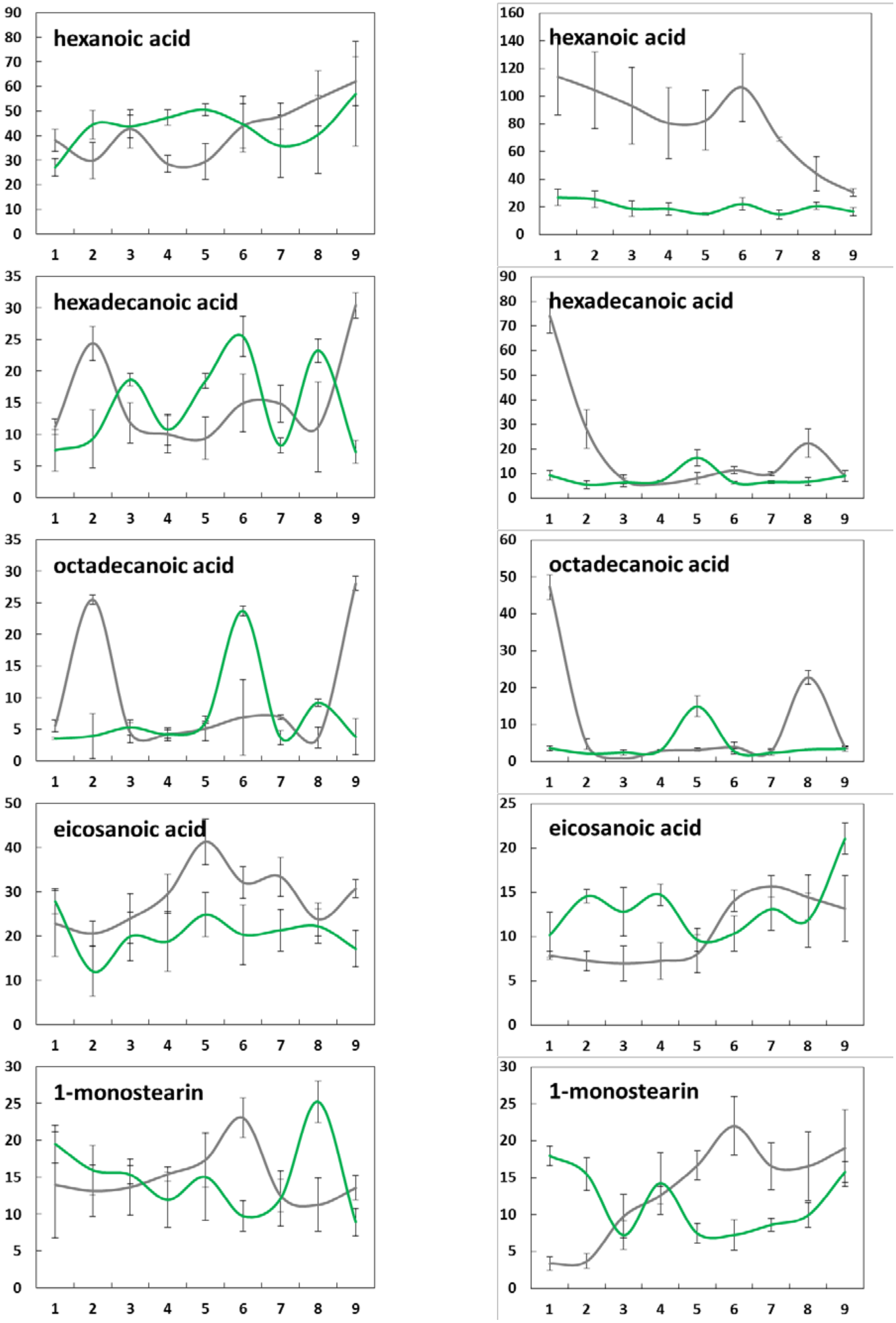

Supplementary Figure 1 – Fatty Acids and Phosphates (2)

Buds **Needles**

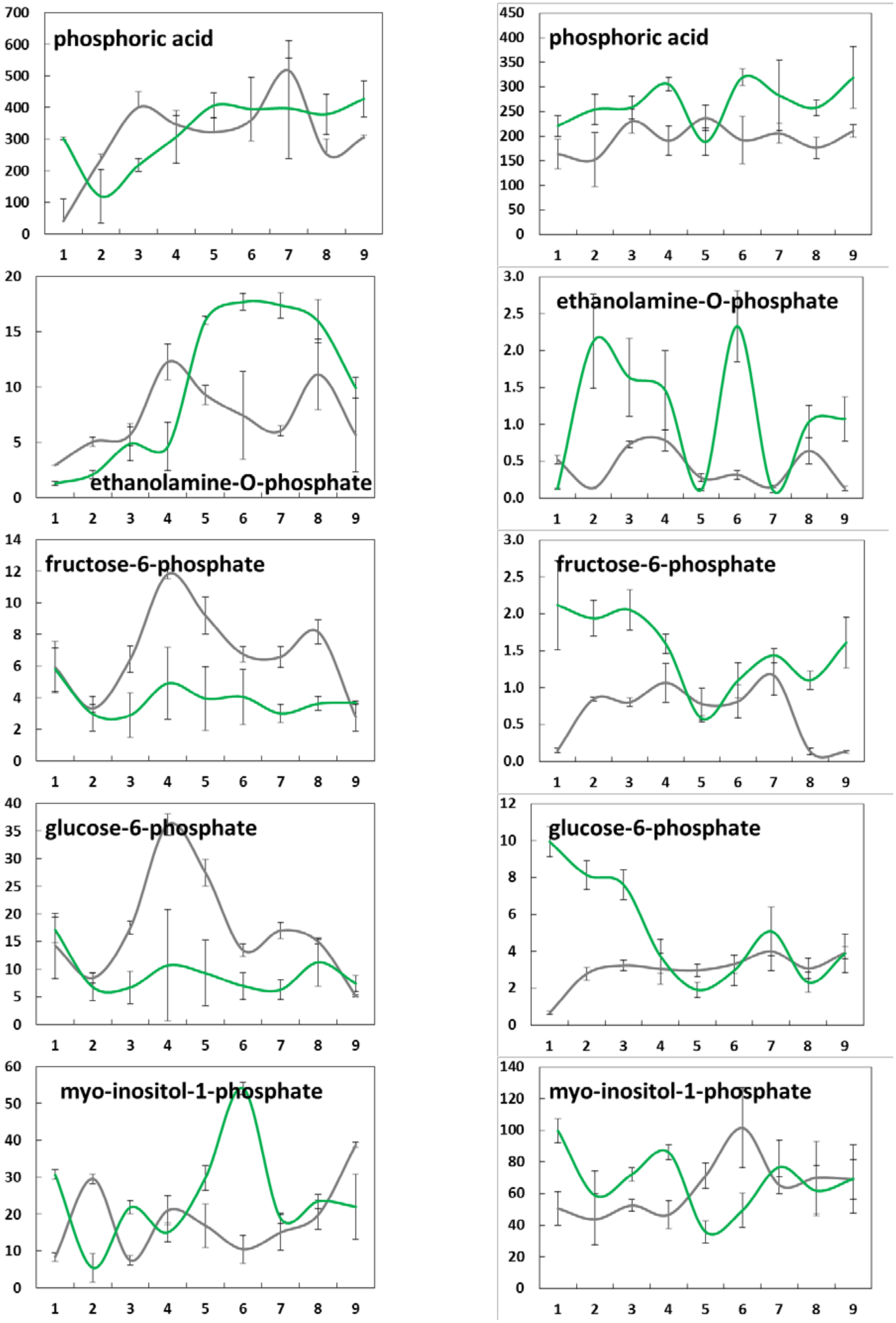

Supplementary Figure 1 – Acids and Alcohols (1)

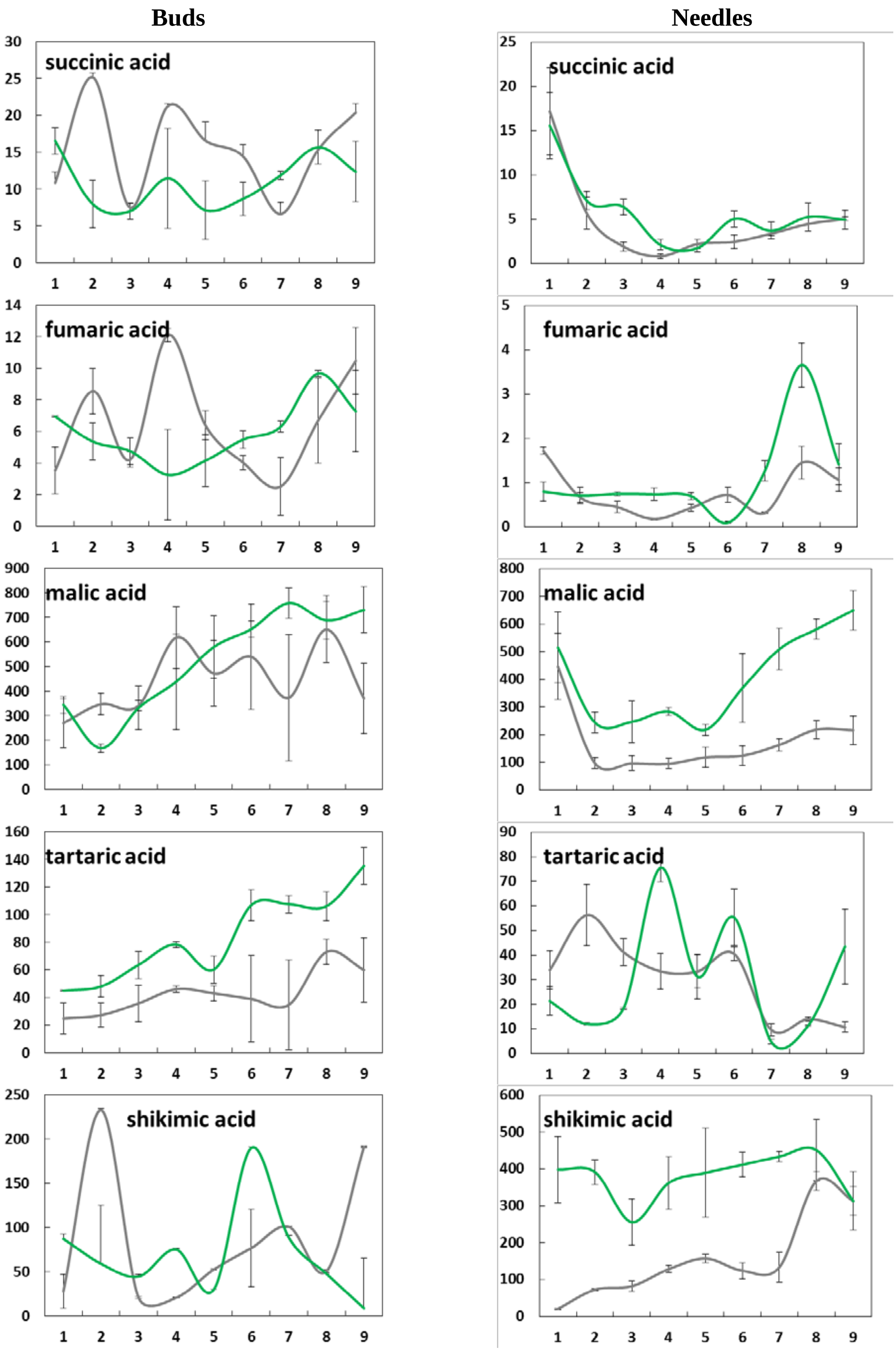

Supplementary Figure 1 – Acids and Alcohol (2)

Buds **Needles**

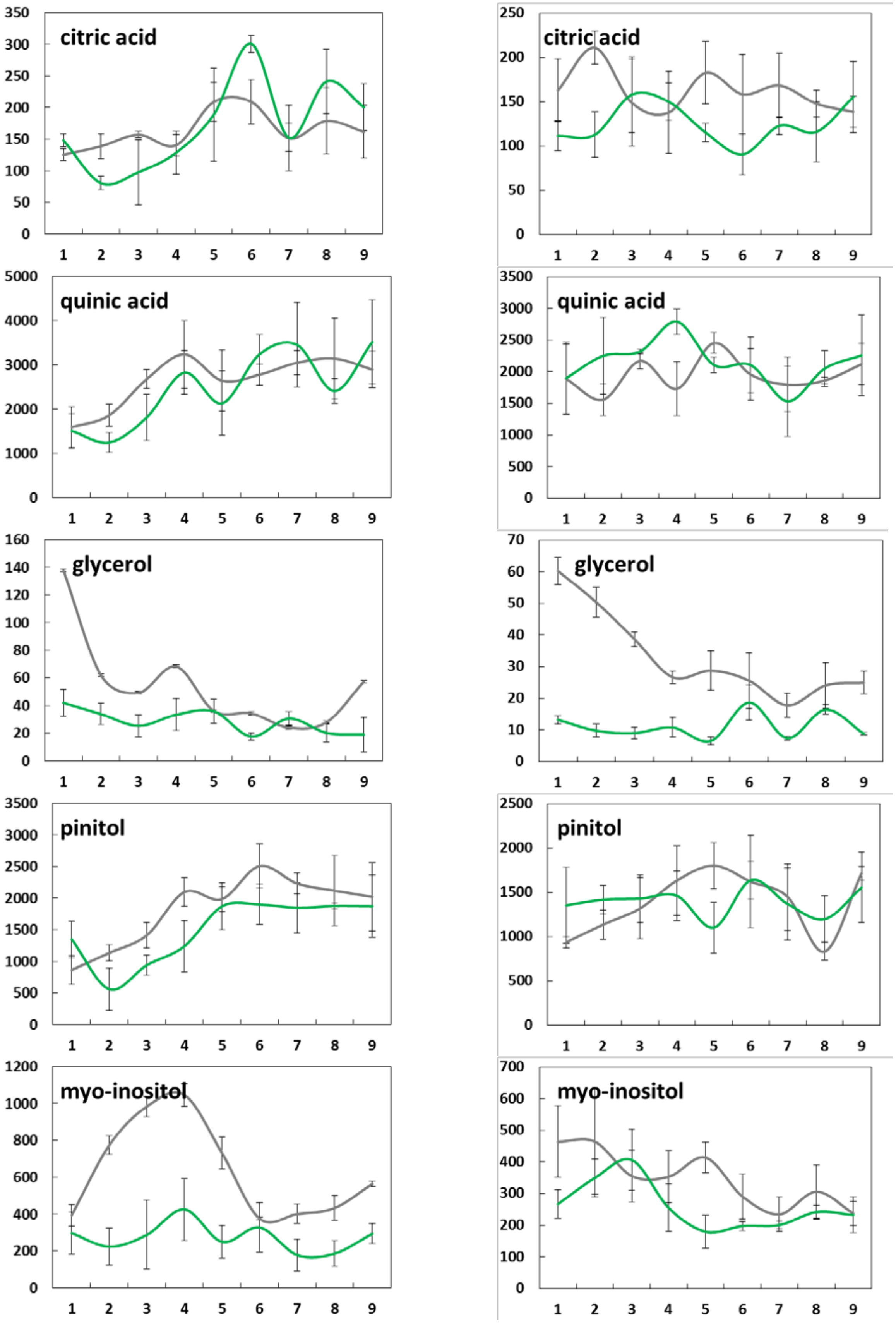

Supplement: Supplementary file 1 [file Image1.PDF]
