# Supplementary material for: Metabolite changes in conifer buds and needles during forced bud break in Norway spruce (Picea abies) and European silver fir (Abies alba)
Source: Front Plant Sci. 2014 Dec 11;5:706. doi: 10.3389/fpls.2014.00706 (PMC4263092; doi:10.3389/fpls.2014.00706)

Supplementary Figure 2. Distance correlation heat maps based on a total of 80 identified metabolites in samples (all replicates; n=3) of either buds (Supplementary Figure **2A**) or needles (Supplementary Figure **2B**) from greenhouse-incubated twigs of both species (*Abies alba* and *Picea abies*) over a 9-weeks period. Orange colours indicate a positive correlation between different metabolites, while bluish colors indicate negative metabolite correlation.

Supplementary Figure 2A – BUDS

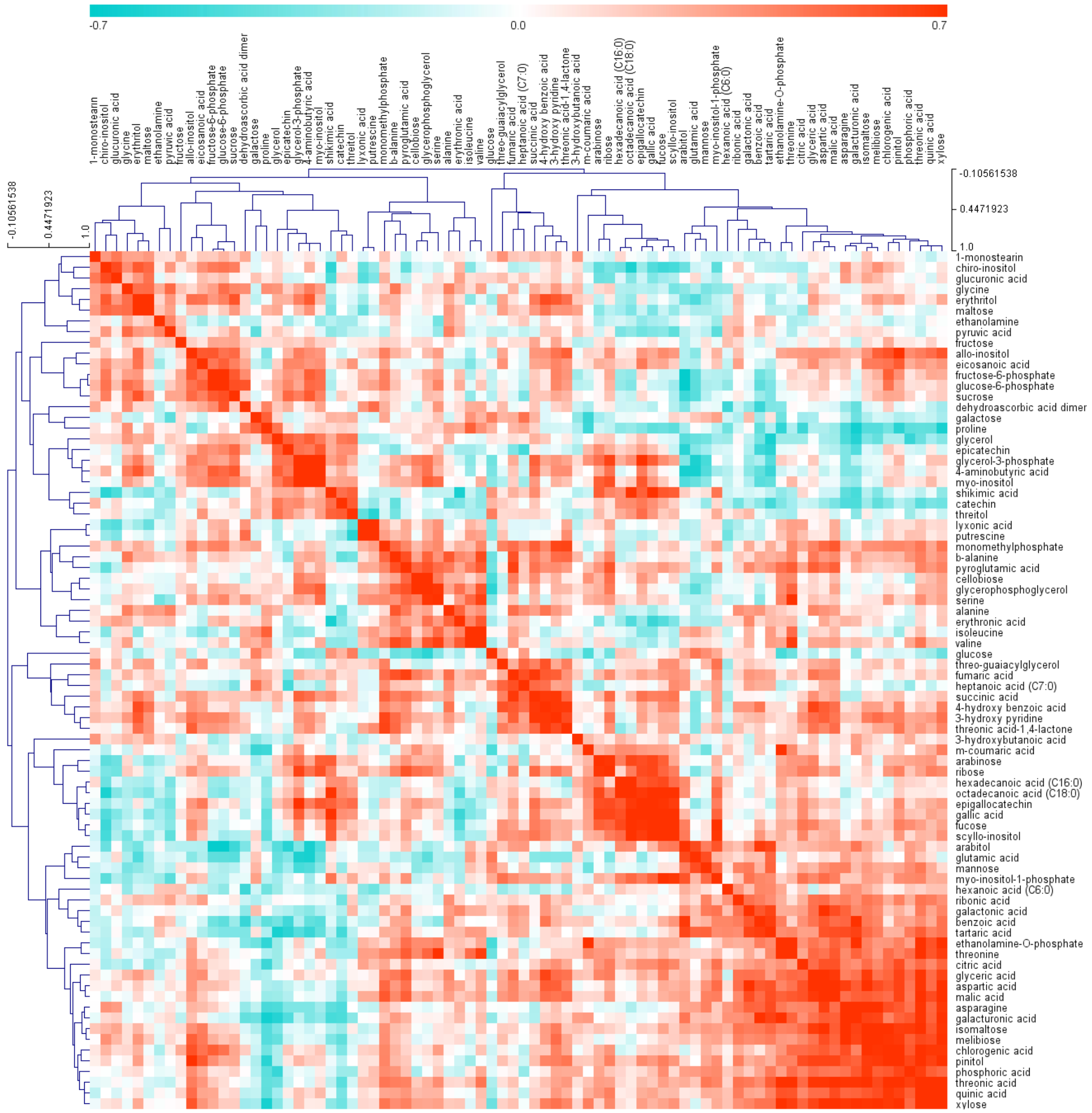

Supplementary Figure 2B – NEEDLES

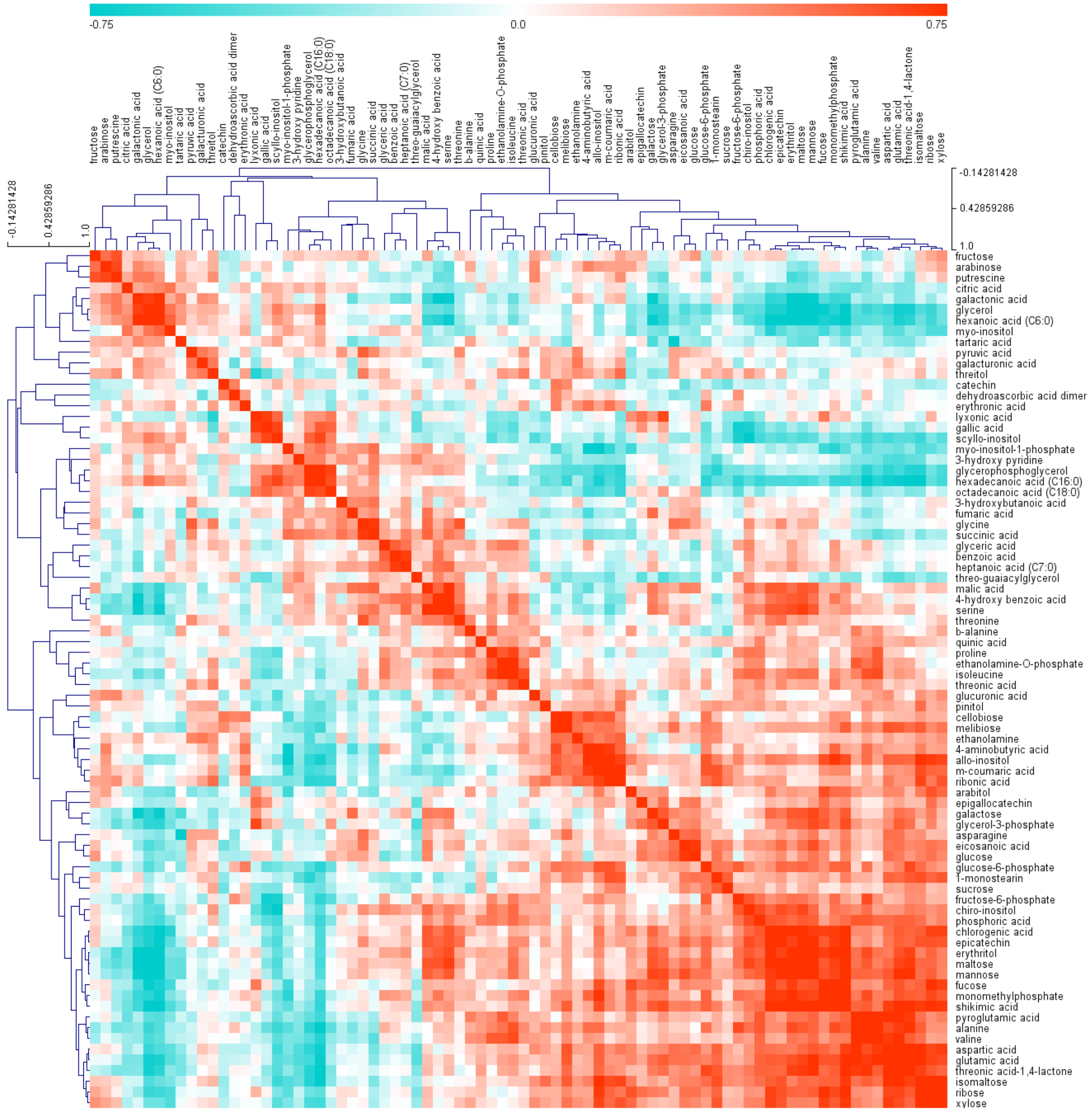

Supplement: Supplementary file 2 [file Image2.PDF]
