# Supplementary material for: Metabolite changes in conifer buds and needles during forced bud break in Norway spruce (Picea abies) and European silver fir (Abies alba)
Source: Front Plant Sci. 2014 Dec 11;5:706. doi: 10.3389/fpls.2014.00706 (PMC4263092; doi:10.3389/fpls.2014.00706)

**Supplementary Figure 3 (A-D).** Volcano scatter plots based on a total of 80 identified metabolites detected in all samples (n=3) of either buds or needles from greenhouse-incubated twigs. Log2(FC) fold change values of metabolite concentration at 9-weeks vs. mean of all time points were plotted against –log10(p) p-values of time points. Metabolites showing a 1.3× increase/ decrease are highlighted: ● $p \leq 0.05$; ● $p \leq 0.01$; ● $p \leq 0.001$

**Supplementary Figure 3A**

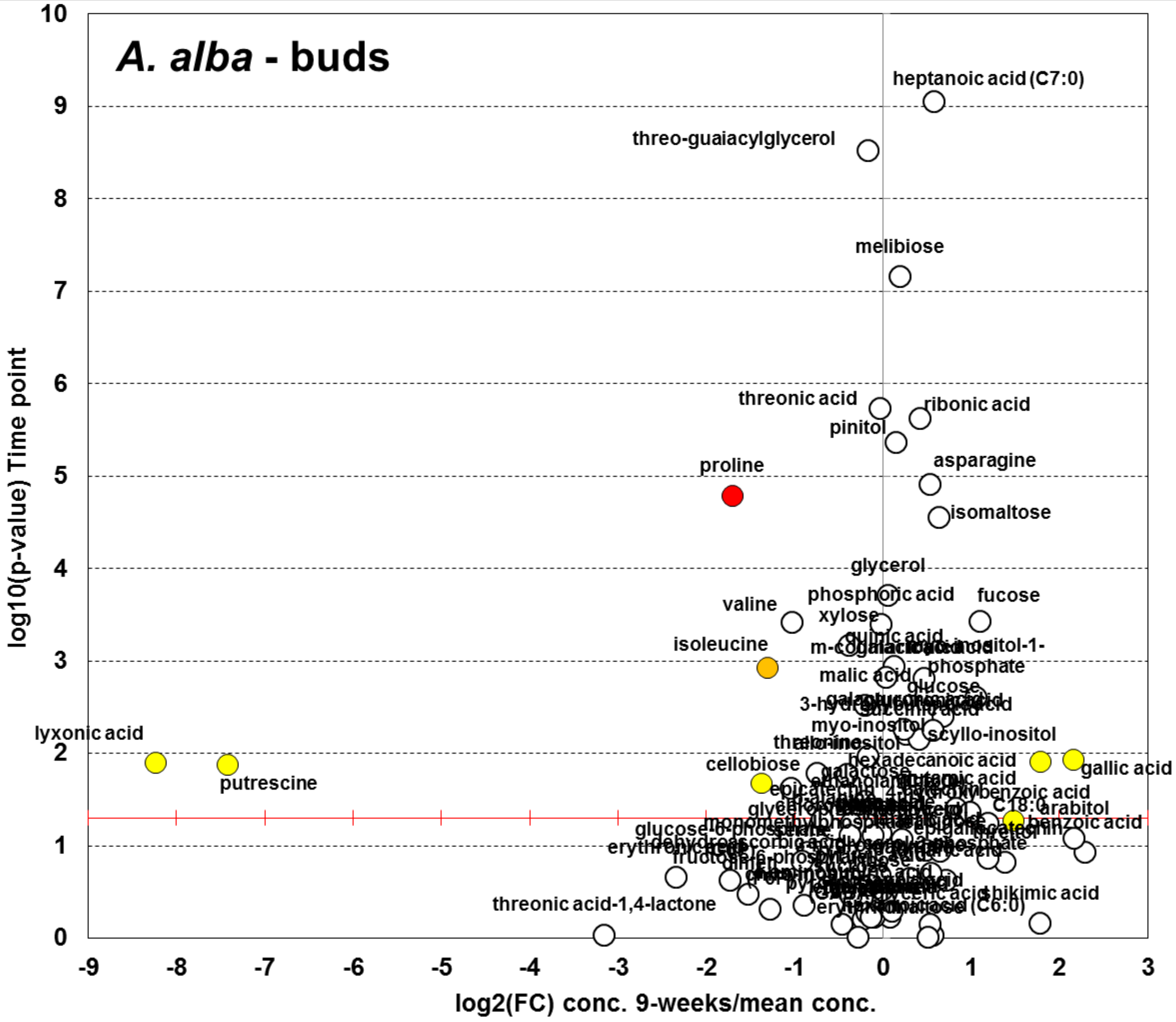

**Supplementary Figure 3B**

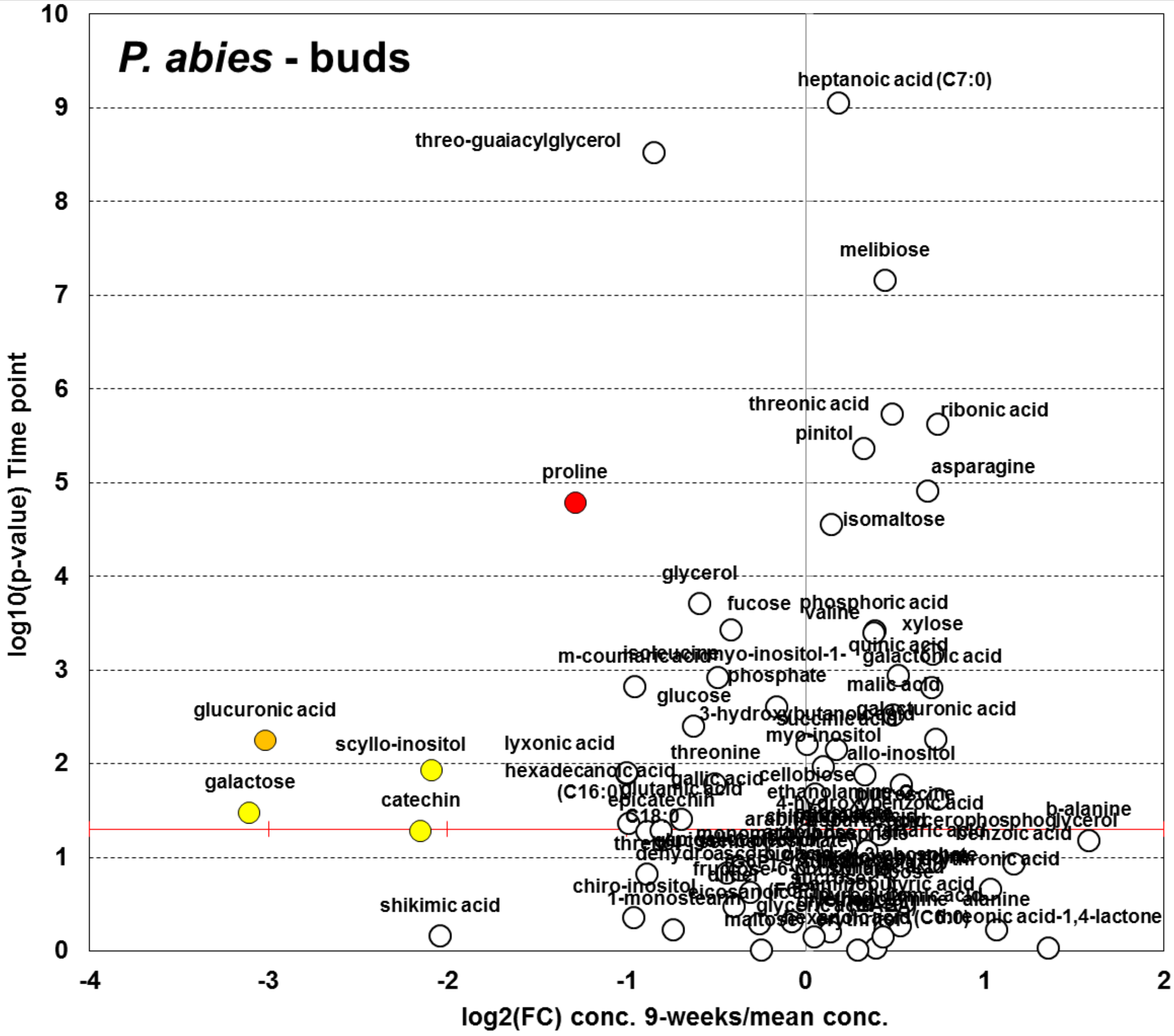

**Supplementary Figure 3C**

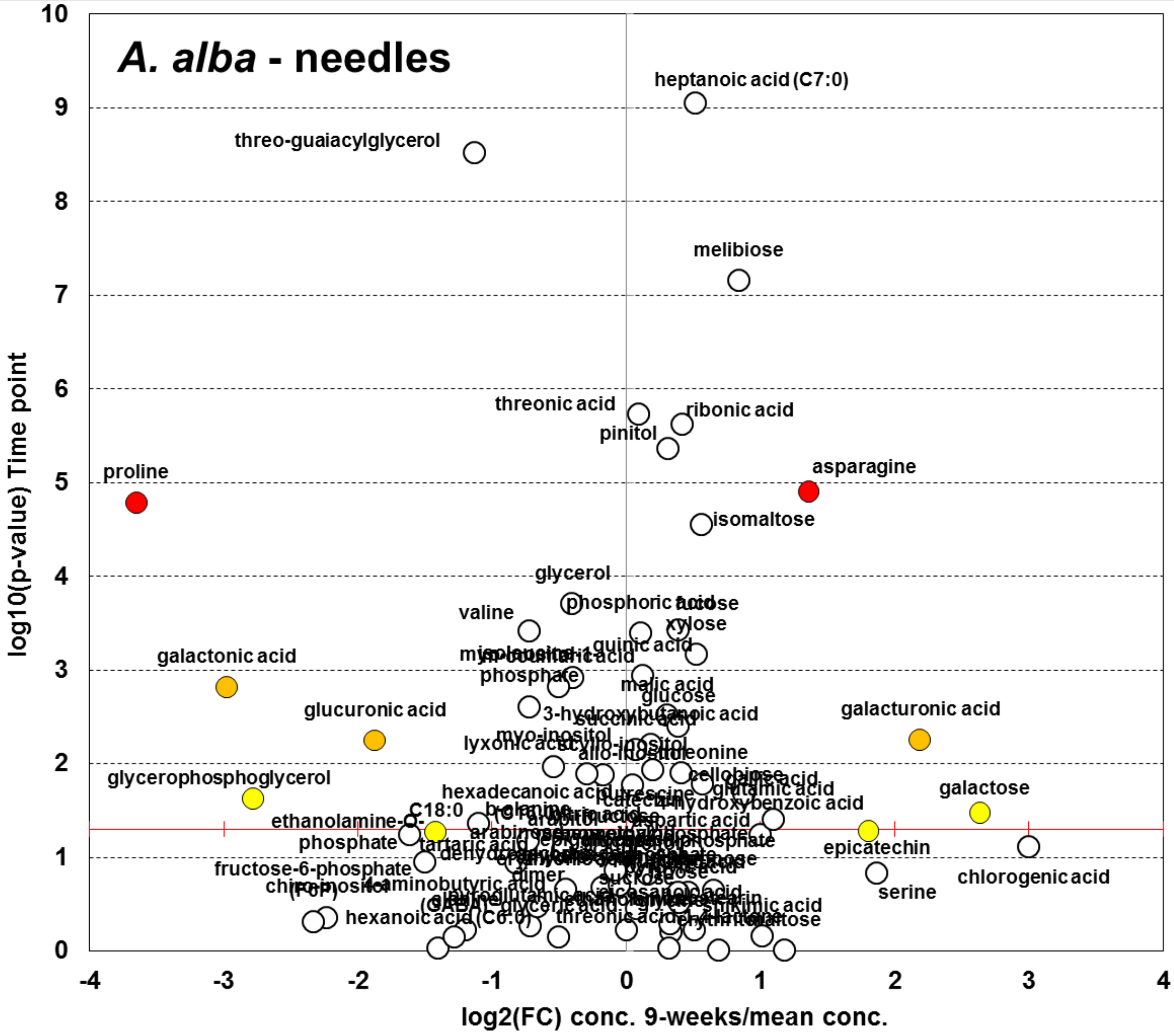

**Supplementary Figure 3D**

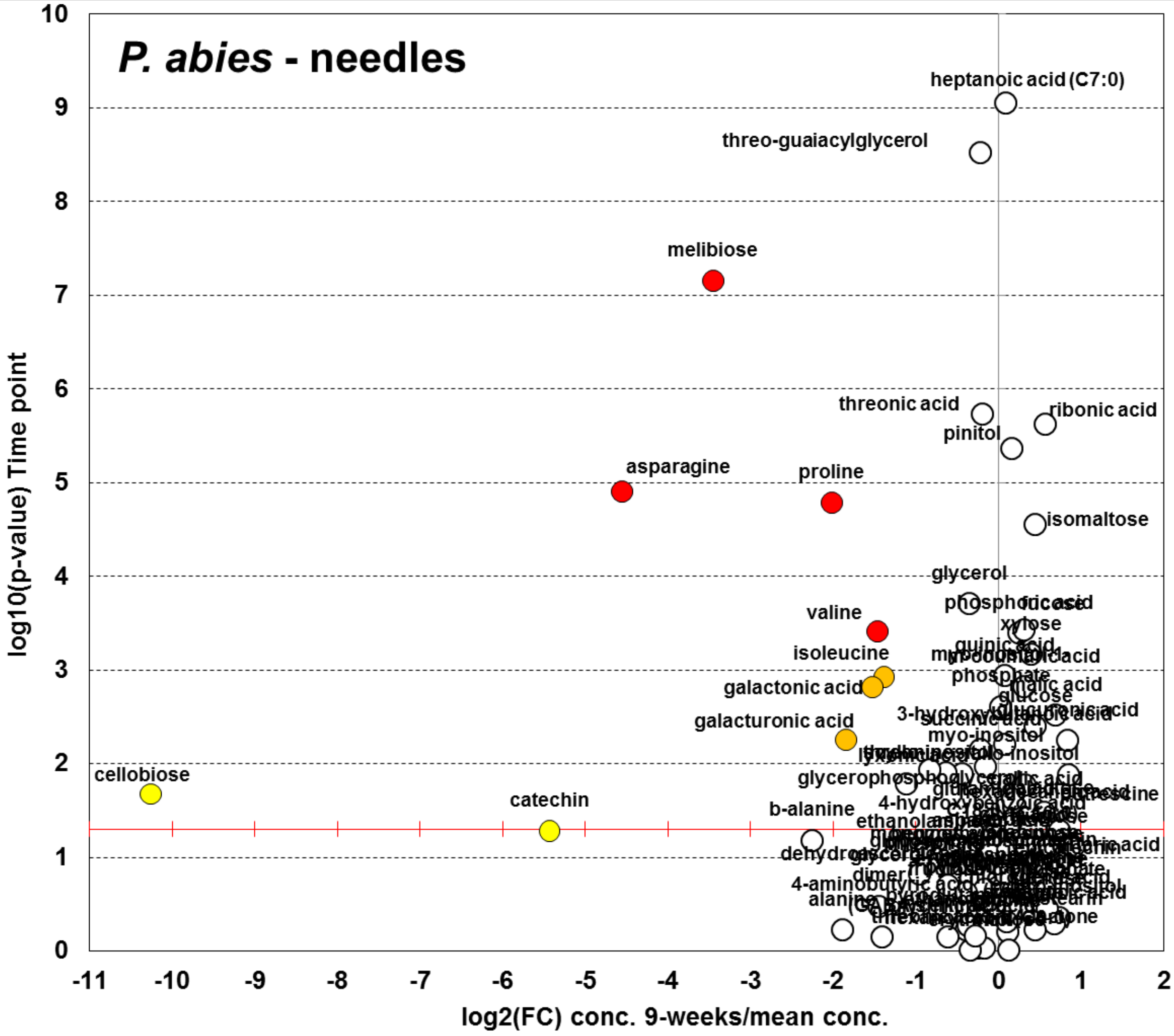

Supplement: Supplementary file 3 [file Image3.PDF]
